# Organelle-targeting ratiometric fluorescent probes: design principles, detection mechanisms, bio-applications, and challenges

**DOI:** 10.1039/d3sc01036h

**Published:** 2023-05-12

**Authors:** Manoj Kumar Goshisht, Neetu Tripathi, Goutam Kumar Patra, Manohar Chaskar

**Affiliations:** a Department of Chemistry, Natural and Applied Sciences, University of Wisconsin—Green Bay 2420 Nicolet Drive Green Bay WI 54311-7001 USA Kumarm@uwgb.edu; b Department of Chemistry, Government Naveen College Tokapal Bastar Chhattisgarh 494442 India mkgh07@gmail.com; c Department of Chemistry, Guru Nanak Dev University Amritsar Punjab 143005 India neetutripathi1990@yahoo.com neetuchem1990@gmail.com; d Department of Chemistry, Faculty of Physical Sciences Guru Ghasidas Vishwavidyalaya Bilaspur Chhattisgarh 495009 India patragoutam137@gmail.com; e Department of Technology, Savitribai Phule Pune University Ganeshkhind Pune 411007 India chaskarmanohar@gmail.com

## Abstract

Biological species, including reactive oxygen species (ROS), reactive sulfur species (RSS), reactive nitrogen species (RNS), F^−^, Pd^2+^, Cu^2+^, Hg^2+^, and others, are crucial for the healthy functioning of cells in living organisms. However, their aberrant concentration can result in various serious diseases. Therefore, it is essential to monitor biological species in cellular organelles such as the cell membrane, mitochondria, lysosome, endoplasmic reticulum, Golgi apparatus, and nucleus. Among various fluorescent probes for species detection within the organelles, ratiometric fluorescent probes have drawn special attention as a potential way to get beyond the drawbacks of intensity-based probes. This method depends on measuring the intensity change of two emission bands (caused by an analyte), which produces an efficient internal referencing that increases the detection's sensitivity. This review article discusses the literature publications (from 2015 to 2022) on organelle-targeting ratiometric fluorescent probes, the general strategies, the detecting mechanisms, the broad scope, and the challenges currently faced by fluorescent probes.

## Abstract 2

Biological species, including reactive oxygen species (ROS), reactive sulfur species (RSS), reactive nitrogen species (RNS), F^−^, Pd^2+^, Cu^2+^, Hg^2+^, and others, are crucial for the healthy functioning of cells in living organisms.

## Graphical abstract

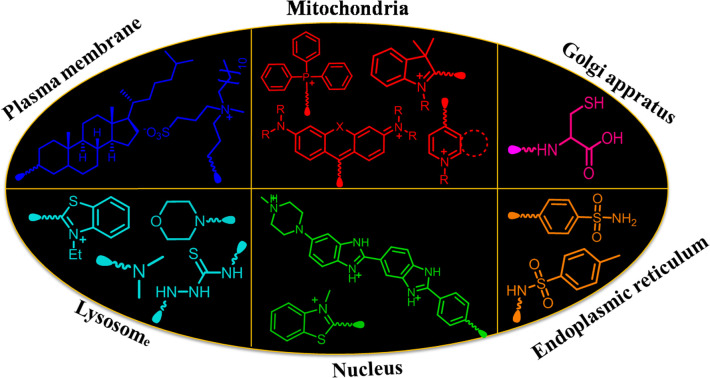

## 1 Introduction

In this modern era, everyone is interconnected. Similarly, in living beings, cells, organelles and biological species are interconnected and act cooperatively for the normal functioning of living systems.^1,2^ For instance, biological species (anions, cations, thiols, nitrogen and oxygen) perform a variety of role in organelles such as signaling molecules, enzyme cofactors, *etc.* However, their chemical imbalance can cause cellular malfunction.^3^ Therefore, smart research work on organelles and biological species is essential, which is challenging.

Over the years, significant studies and research work have been done on organelles and biological species present within the organelles. For example, organelle-targeting medicine has been developed for curing various diaeases.^4,5^ Bioimaging technique has been developed for examining biomolecules in living cells and tissues.^6^ Choi *et al.* studied fluorescent probes for organelles with respect to recent advances and bio-applications.^7^

Nowadays, fluorescent probes for targeting organelles have gained more attention due to their excellent photo-physical characteristics, high sensitivity, rapid response, low cost, non-invasiveness, and real-time imaging.^8^ Notably, many single emission probes are unable to withstand environmental interference. Through a built-in correction of two emissions at different wavelengths, ratiometric fluorescence probes could eliminate background interference.^9^ Additionally, ratiometric fluorescence imaging has an even better resolution due to the two clearly defined emission peaks.^10^ Therefore, the development of ratiometric fluorescent probes is a winning strategy for the sensitive detection of small molecules, thanks to its reduced environmental effect.

Although there are several outstanding reviews in the literature based on ratiometric probes with sensing applications, each one has concentrated on a single aspect, such as the optical processes, a particular class of fluorophores, or a specific subset of target analytes. In 2018, Huang *et al.* reported ratiometric optical nanoprobes for molecular detection and imaging.^10^ In an excellent review, the design principles and applications of fluorescent probes in the ratiometric detection of anions, cations, and biological molecules has been beautifully demonstrated.^11^ Another review article highlighted various chemo/probe-based semiconductor quantum dots (QDs).^12^

In 2020, Wu *et al.* summarized recent advancements in the metal–organic framework (MOF)-based ratiometric fluorescent probes.^13^ Recently, Bigdeli *et al.* published a review article on fluorescent nanoprobes for visual detection.^8^

We illustrate the design principles, fundamental detection mechanisms, and applications of organelle-targeting ratiometric fluorescent probes. The current limitations and prospective future directions, which would spur additional research interest and bring up fresh opportunities for biological analysis, are also covered.

## 2 Design principles

In designing fluorescent probes, photo-physical parameters such as photo-induced electron transfer (PET), internal charge transfer (ICT), monomer–excimer formation, Förster resonance energy transfer (FRET), and excited state intramolecular proton transfer (ESIPT) are frequently used. FRET is a relatively excellent strategy for designing ratiometric fluorescent probes and increasing the Stokes shift.^14^

**Fig. 1:**
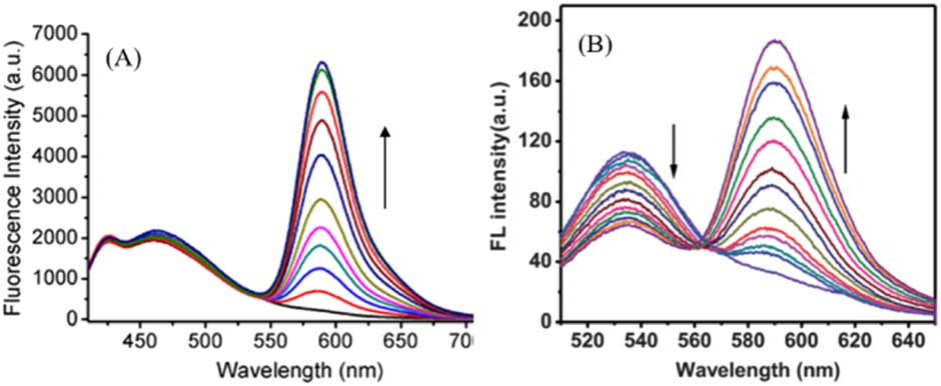
Schematic representation of the ratiometric fluorescence change when (A) static + dynamic fluorophores (reprinted from ref. 15, copyright 2019, Elsevier) and (B) dynamic + dynamic fluorophores (reprinted from ref. 17, copyright 2012, The Royal Society of Chemistry) are utilized.

Generally, the design of ratiometric probes involves the combination of two fluorophores, one reference fluorophore (may or may not show a change in emission intensity upon interaction with the analyte) and another dynamic fluorophore (always shows a change in emission intensity upon interaction with the analyte).^8^ Generally, ratiometric changes in the emission spectrum are of the following type:

(I) Static + dynamic change: herein, upon the addition of an analyte, the emission intensity of one fluorophore is almost kept unchanged, whereas other fluorophores may undergo an increase/decrease/shift in emission intensity (Fig. 1A).^15,16^

(II) Dynamic + dynamic change: herein, both the fluorophores change (increase/decrease/or shift in emission intensity), but in opposite directions (Fig. 1B).^17^

**Fig. 2:**
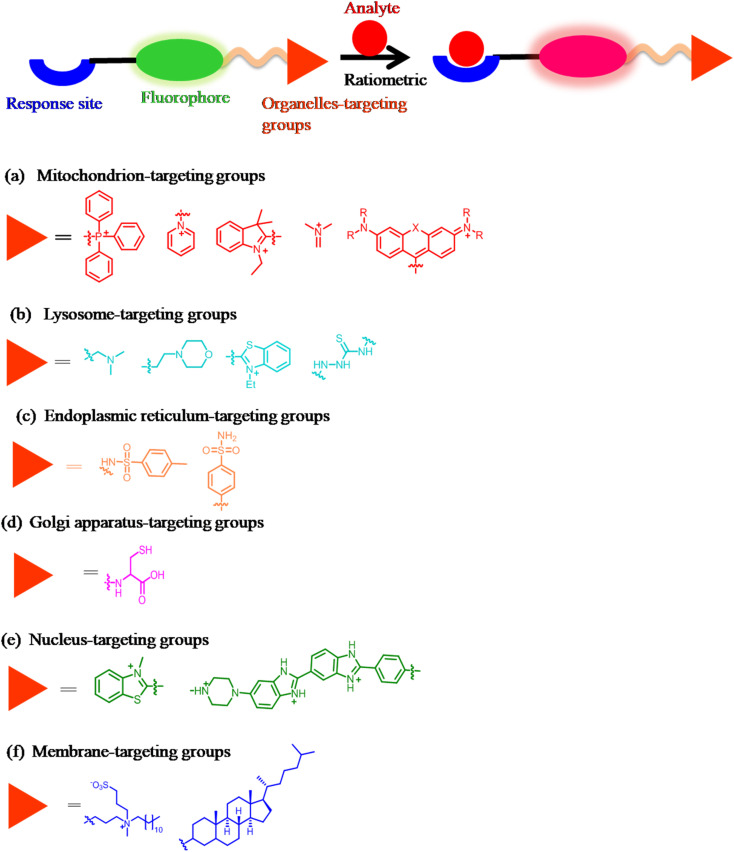
General strategies for designing organelle-targeting ratiometric fluorescent probes and common organelle-targeting groups for (a) mitochondria; (b) lysosome; (c) endoplasmic reticulum; (d) Golgi apparatus; (e) nucleus; (f) membrane.

Generally, organelle-targeting ratiometric fluorescent probes contain a fluorophore, recognition units, and targeting moieties (Fig. 2). The organelle-targeting groups are selective to specific organelles. For example, due to its alkalinity, the morpholine group is a conventional lysosome-targeting moiety.^18^ In addition, the polarity-dependent approach helps create various biological probes, which are helpful for imaging multiple organelles.^19^

### 2.1 Designing mitochondria-targeting ratiometric fluorescent probes

Since the mitochondrial membrane typically has a potential of −180 mV, this property is employed to direct lipophilic positively charged probes into the mitochondria.^20^ The common mitochondrial-targeting units include the quaternized pyridine moiety,^21^ triphenylphosphonium (TPP),^22,23^ indole,^24^ cyanine,^25,26^ pyridinium,^27,28^ and rhodamine^29^ (Fig. 2a).^30–33^ Furthermore, to ascertain the mitochondria localization of probes in cells, the co-localization experiment was performed using commercially available mitochondrion-specific dyes such as MitoTracker Green FM, MitoTracker Red, and MitoTracker Orange.^30^ Generally, mitochondrion-targeting fluorescent probes consist of a fluorophore linked to the mitochondrion-targeting moiety and an activation unit.

### 2.2 Designing lysosome-targeting ratiometric fluorescent probes

Modifying them with lipophilic amines is the most popular method for directing probes into lysosomes.^34–36^ Due to the membrane-impermeable protonated amines in lysosomes, selective probe trapping occurs. The pyridine group,^37^ monothio-bishydrazide moiety,^14^ and morpholine^15^ are the common lysosome-targeting groups (Fig. 2b). Typically, the design of the probe for lysosome requires linking of the fluorophore with the lysosome-targeting group and an activation unit.

### 2.3 Designing endoplasmic reticulum-targeting ratiometric fluorescent probes

The commonly used ER-targeting moieties include glibenclamide,^38^ methyl sulphonamide,^39^ and the *p*-toluenesulfonamide group (Fig. 2c).^40,41^ ER-targeting fluorescent probes generally have (1) a moderate size (conjugated band numbers (CBN < 40)), (2) a cationic character, and (3) an appropriate lipophilicity (+6 > log *P*_oct_ > 0).^42^ The ER-targeting fluorescent probes mainly track cellular concentrations of stress-responsive substances like NO, H_2_S, H_2_O_2_, and HOCl.^43,44^

### 2.4 Designing Golgi-apparatus-targeting ratiometric fluorescent probes

Motivated by the abundance of cysteine residues in the Golgi apparatus, Huang and coworkers proved l-cysteine as an effective Golgi apparatus targeting ligand. They created various probes using this technique (Fig. 2d).^45–48^

### 2.5 Designing nucleus-targeting ratiometric fluorescent probes

The nuclear envelope is a highly controlled membrane barrier. Therefore, passive diffusion or active transport uses the nuclear pore complex (NPC) to target the nucleus.^49^ Small fluorescent probes with cationic centers and hydrophobic planar aromatic structures can selectively label DNA molecules by focusing on the minor grooves in DNA (negatively charged double strands). Some of them have been made available for purchase.^50,51^ From a molecular docking experiment, Ma *et al.* demonstrated electrostatic binding between a positively charged probe and negatively charged nucleus RNA major groove (affinity energy = −5.78 kcal mol^−1^).^52^ Nucleus-targeting unit functionalization is an excellent strategy for delivering fluorescent functional probes into the nucleus of living cells (Fig. 2e).^53,54^

### 2.6 Designing membrane-targeting ratiometric fluorescent probes

Currently, the available probes for the membrane share a common approach: the conjugation of an environment-sensitive fluorophore to generate membrane-specific signals and a membrane-anchoring moiety to minimize the diffusion of the probe (Fig. 2f).^7^

## 3 Detection mechanism

**Fig. 3:**
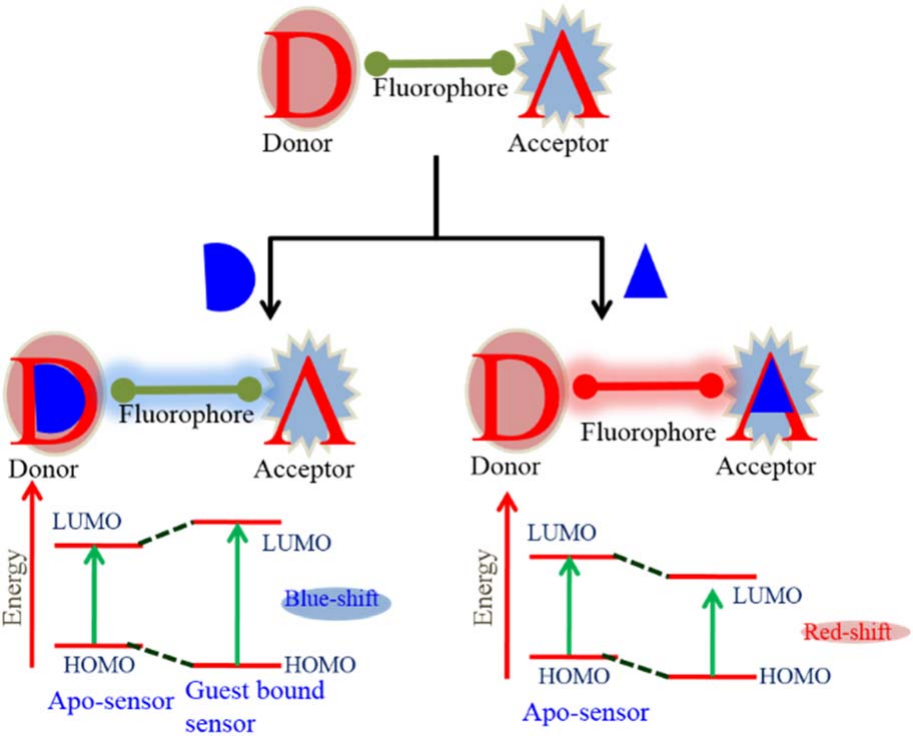
Schematic representation of the internal charge transfer (ICT)-based sensing mechanism in ratiometric design.

**Fig. 4:**
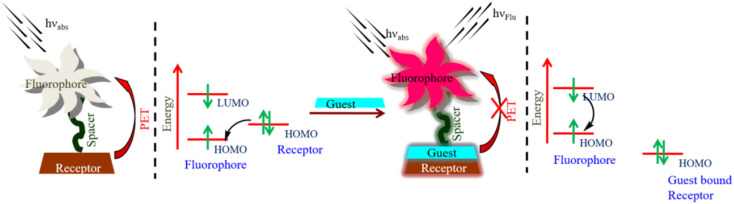
Schematic representation of the photo-induced electron transfer (PET)-based sensing mechanism in ratiometric design.

**Fig. 5:**
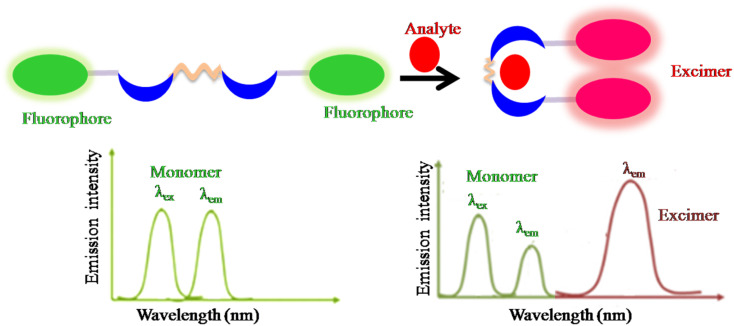
Schematic representation of the monomer–excimer formation-based sensing mechanism in ratiometric design.

**Fig. 6:**
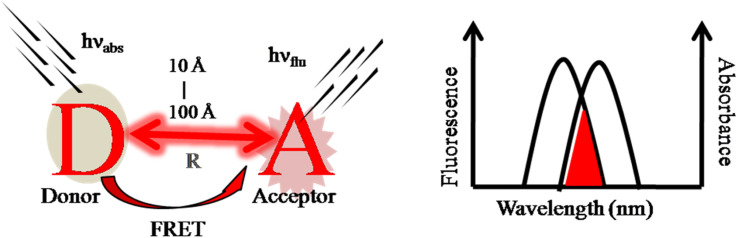
Schematic representation of the Förster resonance energy transfer (FRET)-based sensing mechanism in ratiometric design.

**Fig. 7:**
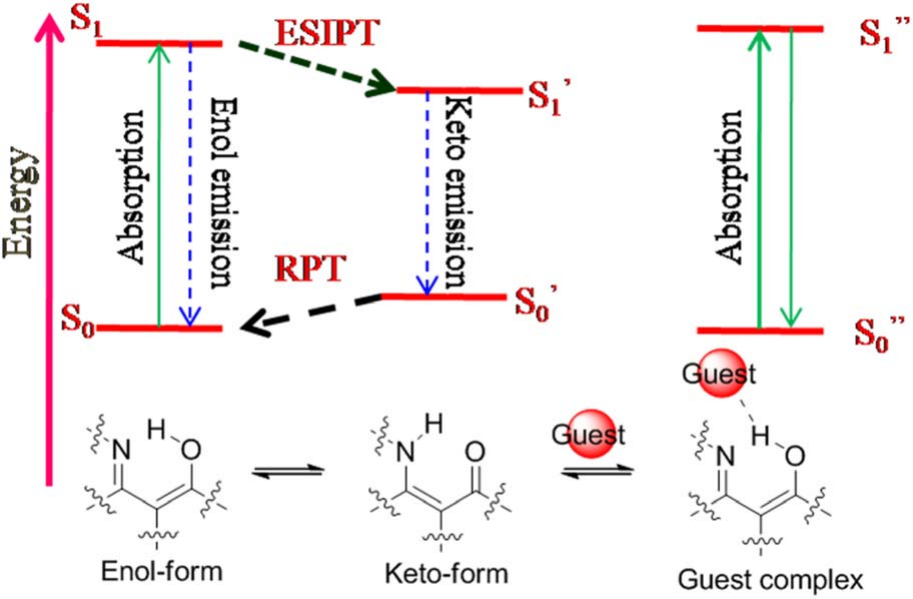
Schematic representation of the excited state intra-molecular proton transfer (ESIPT)-based sensing mechanism in ratiometric design.

The various fluorescence-based sensing mechanisms include photo-induced electron transfer (PET), internal charge transfer (ICT), monomer–excimer formation, Förster resonance energy transfer (FRET), and excited state intra-molecular proton transfer (ESIPT). These mechanisms are available in detail in our recent publications^55–57^ and some excellent reviews.^9,58^ In this section, we explain the fluorescence-based sensing mechanisms briefly. The HOMO localizes on the donor moieties in the ICT-based fluorescent probe. The LUMO is centered on acceptor moieties, thus creating a solid dipole with a charge transfer phenomenon upon excitation. The preferential interaction of the analyte at either the donor or acceptor results in a change in dipole strengths and, consequently, spectral shifts (Fig. 3). To effectively measure ratiometrically, an ICT-based probe should exhibit a visible difference in fluorescence intensity as well as a significant change in emission wavelength. In the PET process (Fig. 4), upon excitation, an electron is transferred from the HOMO (highest occupied molecular orbital) of the receptor (donor) to the LUMO (lowest unoccupied molecular orbital) of the fluorophore (acceptor). However, in the case of the guest-bounded receptor, the HOMO energy levels become lower than those of the fluorophore, inhibiting the PET process and fluorescence change. In the monomer–excimer-based sensing mechanism, upon the addition of an analyte, generally excited state complex formation (excimer) occurs by the interaction between the excited states of one fluorophore and the ground state of another molecule (Fig. 5). The probes that include polyaromatic hydrocarbon (PAH) moieties, such as pyrene, anthracene, *etc.*, typically display this type of sensing mechanism. In the FRET process, energy is transferred from the excited donor molecule to the ground-state acceptor molecule. The critical parameter which governs the FRET phenomenon is spectral overlaps (donor emission spectrum and acceptor absorption spectrum) (Fig. 6). FRET-based probes prove to be an excellent tool for ratiometric imaging due to the stoichiometric relationship between the donor (D) and acceptor (A). In the ESIPT process, proton transfer occurs from the preferred enol-form to the excited state keto-form upon excitation. During relaxation, the excited state keto-form converts back to the enol form by reverse proton transfer. Interestingly, intense fluorescence, large Stokes-shift, and photo-stability are the various unique features of ESIPT-based probes (Fig. 7).

## 4 Mitochondria-targeting ratiometric fluorescent probes

Mitochondria are organelles that play a vital role in cell physiology, including oxidative respiration, ATP production, and signal transduction. Mitochondria are widely known as the main generators of various ROS, RSS and RNS. Thus, probes that can specifically target mitochondria play a key role in monitoring multiple functions of mitochondria and mitochondrion-related illnesses.^59–62^

### 4.1 Reactive oxygen species (ROS) detection in mitochondria

The reactive oxygen species H_2_O_2_ and HOCl are potent oxidants with antibacterial capabilities.^63,64^ However, the aberrant synthesis of ROS species *in vivo* has been linked to several illnesses, including lung damage, atherosclerosis, osteoarthritis, and rheumatoid arthritis.^27^ Therefore, real-time and on-site detection of ROS is an exciting research topic.

**Fig. 8:**
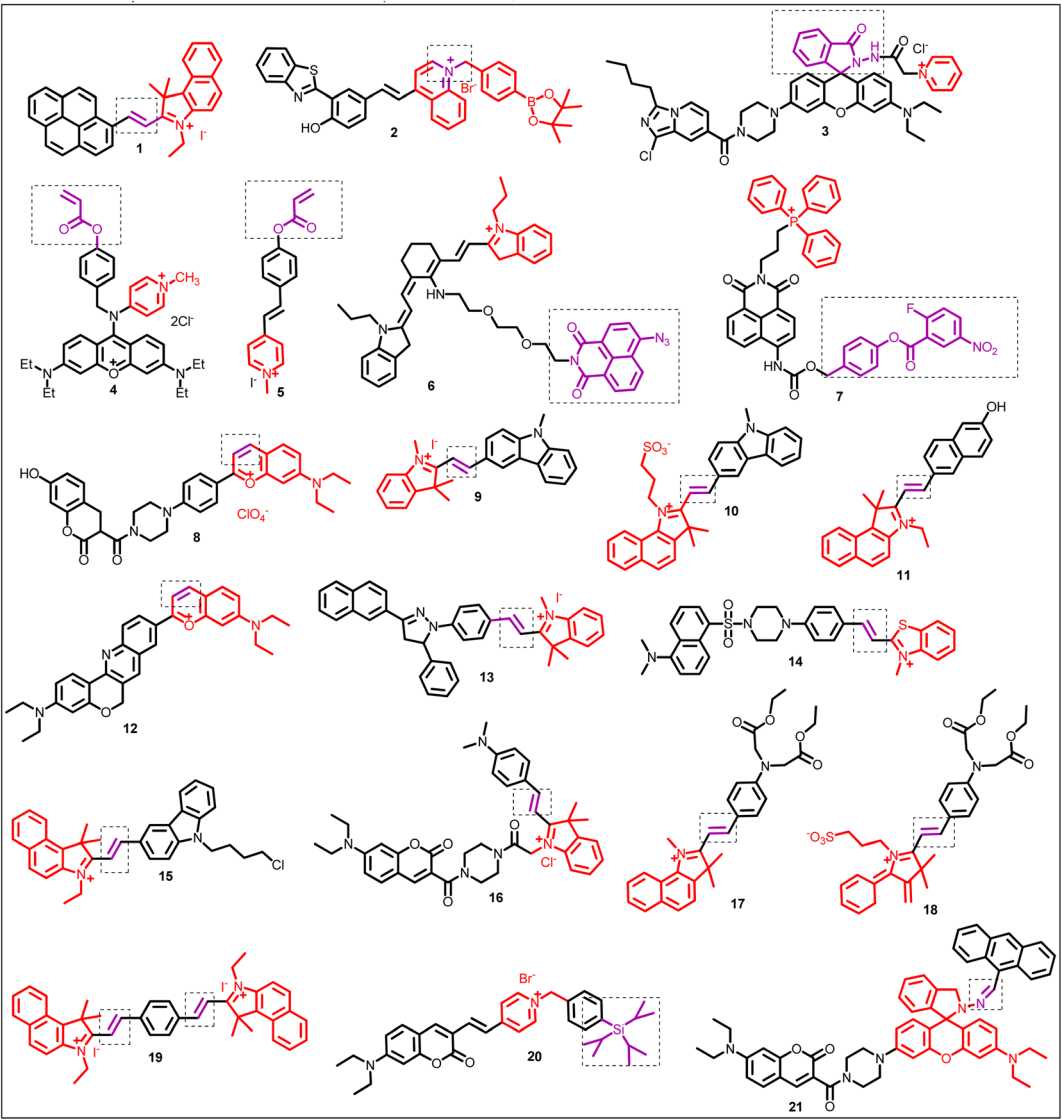
Chemical structure of mitochondrion-targeting ratiometric fluorescent probes (1–21) (red color: mitochondrion-targeting unit; purple color with dotted box: response site).

Hu *et al.* constructed probe 1 with far-red emission by combining a pyrene unit (electron donor, high quantum yield, ability to form a complex in the excited state) with a benzo[*e*]indolium unit (electron acceptor, extended π-conjugation, mitochondrion-targeting group) linked by an ethylene bridge (Fig. 8, Table 1).^65^ In EtOH/PBS solution, the free probe exhibited emission at 632 nm (*λ*_ex_ = 525 nm). Upon the addition of ClO^−^, emission at 632 nm gradually fades away with a concomitant increase in fluorescence intensity at 455 nm (blue emission). Probe 1 with excellent mitochondrial targeting features, such as high selectivity (detection limit = 182 nM), fast response time, significant Stokes–Stokes shift (107 nm), photostability, and live cell membrane permeability, displayed potential for detecting ClO^−^ in mitochondria.

**Table 1:** Summary of organelle-targeting ratiometric fluorescent probes

BS	Organelle	Targeting moiety	Analyte	*Λ*_ex_ (nm)	*Λ*_em_ (nm)	Stokes shift (nm)	LOD	Sensing mechanism	Application	Ref.
1	Mitochondria	Benzo[*e*]indolium group	ClO^−^	525	I_632_/I_455_	107	182 nM	Reaction based and ICT process	HOCl investigation in biological samples	65
2	Mitochondria	Positively charged probe	H_2_O_2_	440	I_594_/I_666_	102–254	23.1 nM	ICT and ESIPT mechanisms	H_2_O_2_ detection in living HeLa cells	70
3	Mitochondria	Quaternized pyridine moiety	ClO^−^	370	I_575_/I_467_	—	10.2 nM	FRET process	Mitochondrial OCl^−^ related studies	71
4	Mitochondria	Positively charged fluorophore	Cysteine	514	I_540_/I_605_	90	33.7 nM	Reaction-based	Quantitatively tracking the Cys distributions in mitochondria	21
5	Mitochondria	Positively charged fluorophore	Cysteine	350	I_518_/I_452_	—	—	Reaction based	Monitoring cysteine level in live cells and in living tissue	76
6	Mitochondria	Cationic cyanine moiety	H_2_S	428 and 636	I_530_/I_733_	—	1.31 μM	ICT process and reaction based mechanism	Monitoring mitochondrial H_2_S in living cells	24
7	Mitochondria	Triphenylphosphonium group	H_2_S_*n*_	405	I_550_/I_485_	109	20 nM	Reaction based	To study H_2_S-linked physiological and pathological processes	81
8	Mitochondria	Benzopyrylium structure	SO_3_^2−^	405	I_455_/I_635_	230	0.078 μM	Nucleophilic addition reaction and FRET process	SO_3_^2−^ imaging in biological systems	88
9	Mitochondria	Indolium group	HSO_3_^−^	350	I_490_/I_590_	—	0.15 μM	Nucleophilic addition reaction	Monitoring SO_2_ derivatives in living cells	89
10	Mitochondria	Benzoindole group	SO_2_ derivatives	405	I_463_/I_625_	—	58 nM	Nucleophilic addition reaction	Monitoring SO_2_ derivatives in living cells	90
11	Mitochondria	Cyanine dyes	SO_3_^2−^/HSO_3_^−^	404	I_467_/I_593_	—	3.6 nM	1,4-Addition reaction	Monitoring SO_2_ derivatives in living cells	91
12	Mitochondria	Positively charged fluorophore	NaHSO_3_	405 and 580	I_514_/I_613_	—	103 nM (*λ*_ex_ = 580 nm) and 17 nM	Nucleophilic addition reaction and ICT process	Detection of SO_2_ derivatives in mitochondria under one-photon and two-photo absorption	92
							(*λ*_ex_ = 405 nm), separately			
13	Mitochondria	Hemi-cyanine dyes	SO_2_ derivatives	380 and 558	I_480_/I_640_	—	80 nM	Nucleophilic addition reaction and ICT process	Detection of SO_2_ derivatives in mitochondria	93
14	Mitochondria	Benzothiazolium moiety	Bisulfite	390	I_540_/I_590_		69 nM	Nucleophilic addition reaction and FRET process	Quantitative detection of HSO_3_^−^ in mitochondria	94
15	Mitochondria	Lipophilic cationic dye probe	HSO_3_^−^	322 and 510	I_462_/I_588_	—	10 nM	1,4-Nucleophilic addition reaction	Detection of SO_2_ derivatives in living cells	95
16	Mitochondria	Indole moiety	SO_3_^2−^	430	I_476_/I_589_	—	12.85 nM	Nucleophilic addition reaction, ICT process and FRET effect	SO_3_^2−^ imaging in living cells	96
17 and 18	Mitochondria	Cyanine dye, 1*H*-benzo[*e*]indolium	HSO_3_^−^/SO_3_^2−^	840	I_476_/I_579_ and I_468_/I_586_ for probes 17 and 18, respectively	169 and 166 for probes 17 and 18, respectively	0.20 μM and 0.11 μM for probes 17 and 18, respectively	Nucleophilic addition mechanisms, ICT process	SO_3_^2−^ imaging in living cells	97
19	Mitochondria	Benz[*e*]indole moiety	HSO_3_^−^/SO_3_^2−^	380	I_480_/I_600_	—	0.1 μM	Nucleophilic addition mechanisms, ICT process	SO_3_^2−^ imaging in living cells	98
20	Mitochondria	Pyridinium salt	F^−^	490	I_539_/I_639_	—	12 nM	Si–O bond cleavage and ICT process	F^−^ investigation in biological samples	100
21	Mitochondria	Rhodamine dyes as fluorescent lipophilic cations	Pd^2+^	400 and 550	I_594_/I_472_	—	—	Chelation induced ring opening of rhodamine spirolactam and FRET process	Ratiometric visualization of Pd^2+^ in mitochondria	29
22	Lysosome	Pyridine group	H_2_O_2_	375	I_550_/I_425_	125	12.8 nM	ICT process	Lysosomal H_2_O_2_ detection	37
23	Lysosome	Morpholine group	HOCl	370	I_589_/I_462_	—	10.2 nM	PET process, TBET process and HOCl induced ring opening of rhodamine spirocyclic form	To monitor HOCl changes in lysosome	15
24	Lysosome	Monothio-bishydrazide moiety	HOCl	410	I_580_/I_480_	—	0.66 μM	FRET process	HOCl detection in living cells	14
25	Lysosome	Morpholine	HOCl	440	I_610_/I_535_	—	0.58 μM	ICT process	Lysosomal HOCl detection in living cells and zebrafish	109
26	Lysosome	Morpholine moiety	Bisulfite	480	I_512_/I_704_	—	0.09 μM	1,6-Conjugate addition reaction	Ratiometric fluorescence imaging of lysosomal bisulfite	114
27	Lysosome	Morpholine moiety	Cysteine	510	I_510_/I_685_	—	0.28 μM	1,6-Conjugate addition	Fluorescence imaging of the lysosomal cysteine	117
28	Lysosome	Morpholine	H_2_S	350	I_415_/I_560_	—	0.43 μM	FRET-based	H_2_S detection in lysosome	118
29	Lysosome	Morpholine	Cu^2+^	440	I_580_/I_519_		1.45 nM	FRET process	Lysosomal Cu^2+^ detection in living cells	123
30	Lysosome	Morpholine	Cu^2+^	360 nm	I_552_/I_486_		105 nM	FRET process	Lysosomal Cu^2+^ detection in living cells	125
31	Lysosome	Morpholine	Hg^2+^	480 nm	535 to 595 nm		0.23 μM	PET process	Lysosomal Hg^2+^ detection in living cells	127
32	ER	—	H_2_O_2_	395	I_540_/I_465_	—	38 nM	Reaction based mechanism	H_2_O_2_ detection in ER	131
33	ER	Methyl sulfonamide	HClO	For *λ*_em_ = 554, *λ*_ex_ = 425; for *λ*_em_ = 588, *λ*_ex_ = 500	I_480_/I_554_	—	0.59 μM	Reaction-based	HClO detection in the ER of living cells	39
the	ER	*p*-Toluenesulfonamide group	HClO	380	I_484_/I_533_		0.1 μM	ICT process and reaction-based	HClO detection in the ER of living cells	41
35	ER	—	HOCl	326	I_450_/I_361_	—	3.6 μM	Nucleophilic borono-Dakin oxidation mechanism	Study of ER signaling and function under oxidative stress	18
36	ER		H_2_S	480	I_650_/I_560_	(∼150 nm)	39.1 nM	Reaction based mechanism	H_2_S detection in living cells and zebrafish	144
37	ER	—	SO_2_ derivatives	440	I_534_/I_610_		16.2 nM	FRET process	Fluorescence imaging of HSO_3_^−^/SO_3_^2−^ in living cells	147
38	ER	—	Cu^2+^	410	I_545_/I_480_		1.1 μM and 0.7 μM for Cu^2+^ and Cu^+^, respectively	Copper-promoted hydrolysis	Imaging copper accumulation in the ER of live cells	150
39	ER	*p*-Toluenesulfonamide	pH	405	I_446_/I_527_		Response to pH in the range 5.0–7.2	ICT–PET–FRET mechanism	Quantitative measurement of pH values in endoplasmic reticulum (ER)	40
40	ER	*p*-Toluenesulfonamide	Carboxylesterase 2 detection	380	I_560_/I_414_	—	—	Reaction-based	Carboxylesterase 2 detection in drug-induced acute liver injury	158
41	Golgi apparatus	Phenylsulfonamide group	H_2_O_2_	—	I_560_/I_470_	—	0.20 μM	Reaction-based	To monitor Golgi oxidative stress and novel drug targets	162
42	Golgi apparatus	Sulfanilamide	ONOO^−^	—	I_650_/I_477_	—	—	Reaction-based	To monitor Golgi oxidative stress and to evaluate drug-induced liver injury	163
43	Golgi apparatus	Aminoquinoline derivative	CO	360	I_520_/I_425_	—	41 nM	Pd^0^-mediated Tsuji–Trost reaction	Fluorescence imaging of CO in cells and zebrafish	164
44	Golgi apparatus	4-CF_3_-7-aminoquinoline	Glutathione (GSH)	320	I_510_/I_425_	—	0.49 μM	Reaction-based	GSH detection and organelle-targeted therapy	165
45	Nucleolus	—	SO_2_ and formaldehyde	365	I_550_/I_635_		0.17 μM	FRET process and Michael addition reaction	Living mice imaging	52
46	Nucleus	—	NO	370	I_530_/I_424_		20 nM	Reaction based	Excellent candidate for use as a green fluorescent nucleus probe or a nucleic acid permanent stain	168
47	Nucleus	*N*-Methyl benzothiazole cations	Nucleus viscosity and G-QDNA	340	I_650_/I_407_	—	—	Twisted internal charge transfer (TICT)	Living cells	169
48	Lipid droplets and nucleus	Coumarin unit for lipid droplet and quinolinium unit for nucleic acid	Polarity change in the cellular environment	405	I_470_/I_670_		—	ICT process	Study of ferroptosis and ferroptosis-linked diseases through fluorescence imaging	170
49	Nucleus	Hoechst unit	DNA	345 nm	I_505_/I_450_		72 nM	FRET process	Excellent probe for the monitoring of nucleus DNA damage	171
50 and 51	Cytomembrane	Alkyl side chain of the pyridine salt	SO_2_	415	I_644_/I_486_	—	—	Michael addition	Bio-application in a mice model	178
52	Plasma membrane	CAZ	pH	380 (basic form) and 480 (acidic form)	496 (basic form) and 566 (acidic form)	—	—	—	Imaging and measuring vesicular acidification	182
53	Mitochondria and lysosome	Semi-cyanine unit for targeting mitochondria and morpholine group for targeting lysosome	SO_2_	400	I_530_/I_600_		0.82 μM	FRET process	Simultaneous detection of endogenous SO_2_ in lysosome and mitochondria by one and two-photon modes.	183
54	Mitochondria and lipid droplets (LDs)	Hemi-cyanine to target mitochondria and neutral form of probe 54 to target LDs	pH	380	I_450_/I_580_	—	—	Reaction-based and ICT process	Furthermore, probe 54 was successfully applied for monitoring the pH fluctuation in living cells under different exotic chemicals	19

An important ROS, endogenous H_2_O_2_, functions as a signaling molecule to control various cellular processes, such as cell division, proliferation, and migration.^66,67^ However, high H_2_O_2_ concentrations can harm proteins and nucleic acids, which are strongly related to many disorders such as malignancies, diabetes, and Alzheimer's disease.^68,69^ Therefore, monitoring of H_2_O_2_ concentration is essential. He *et al.* designed benzothiazole dye (adequate stability, large Stokes-shift, large quantum yield, tunable emission) based probe 2 containing an aromatic boronic ester moiety (H_2_O_2_ recognition group) (Fig. 8, Table 1).^70^ The fluorescence titration of probe 2 with H_2_O_2_ revealed an increase in new emission maxima at 594 nm at the expense of emission at 666 nm. The limit of detection was 23.1 nM. The sensing mechanism was the H_2_O_2_ triggered aromatic boronic ester moiety removal. Furthermore, probe 2, with excellent mitochondrial targeting properties, such as significant Stokes shift (152 nm), photostability, and Pearson's colonization coefficient (0.94), displayed potential for detecting H_2_O_2_ in mitochondria.

Shen *et al.* designed probe 3 based on the FRET platform for detecting OCl^−^ (Fig. 8, Table 1).^71^ In the presence of OCl^−^, probe 3 displayed ratiometric fluorescence change, and the plot of intensity ratio (I_575_/I_467_) with the concentration of OCl^−^ was linear in the range of 0 to 5 μM. The limit of detection was 10.2 nM. From the fluorescence imaging experiment, the applied probe 3 successfully examined endogenous OCl^−^ in Murine RAW 264.7 cells.

### 4.2 Reactive sulfur species (RSS) detection in mitochondria

Reactive sulfur species (RSS), such as cysteine (Cys), hydrogen sulfide (H_2_S), and hydrogen polysulfides (H_2_S_*n*_), are produced in large quantities in mitochondria and are associated with critical mitochondrial-related pathological and physiological processes.^7^ Cys can act as an antioxidant in mitochondria by removing various ROS from mitochondria to stop oxidative damage.^72^ Additionally, Cys is necessary for the mitochondrial process of protein turnover.^73–75^ Therefore, it is essential to identify and measure Cys in real-time within cells, particularly in the mitochondria, to understand the pathological and physiological processes properly.

Yang *et al.* designed xanthylene-based fluorescent probe 4 for the ratiometric detection of cysteine levels in the mitochondria (Fig. 8, Table 1).^21^ The probe 4 containing an acryloyl moiety (responsive site for cysteine) and a benzyl group (for easy distribution in the mitochondria) showed strong red fluorescence at *λ*_em_ = 605 nm (*λ*_ex_ = 490 nm). In the presence of cysteine, probe 4 underwent ratiometric fluorescence change with the formation of new emission maxima at 540 nm and the simultaneous decrease in emission intensity at 605 nm. The limit of detection was 33.7 nM for cysteine. Applied probe 4 examined endogenous cysteine levels in HeLa cells through bioimaging, demonstrating potential applications in natural areas.

Considering the advantage of two-photon fluorescence microscopy and ratiometric detection, Niu *et al.* developed probe 5 for detecting cysteine over other biothiols (Fig. 8, Table 1).^76^ In the presence of cysteine, the fluorescence spectrum of probe 5 exhibited ratiometric change (I_518_/I_452_), linear in the range between 0.5 and 40 μM. Interestingly, the merocyanine fluorophore and probe 5 exhibited a large two-photon cross-section (*Φ*_*σ*_max__) of 72.6 GM (*λ*_ex_ = 760 nm) and 65.2 GM (*λ*_ex_ = 740 nm), respectively, favorable in producing bright and high contrast images of living samples. In addition to detecting cysteine in live cells and mitochondria, probe 5 exhibited promising application for monitoring cysteine concentration in living tissues (down to 150 μm depth) using two-photon fluorescence microscopy.

H_2_S plays essential roles in mitochondria, such as scavengers for reactive oxygen species,^77^ and is associated with various mitochondrial-related pathological and physiological processes.^78,79^ Therefore, monitoring of H_2_S level in mitochondria is crucial. Liu *et al.* proposed probe 6 integrated with cyanine (mitochondria-targeting group) and naphthalimide group (responsive to H_2_S) for ratiometric detection of H_2_S in mitochondria (Fig. 8, Table 1).^24^ In the CTAB solution, upon the addition of Na_2_S, probe 6 displayed ratiometric fluorescence response, and the intensity ratio (I_530_/I_733_) showed good linearity in the range of 1–9 μM. The detection limit was 1.31 μM for Na_2_S. In the presence of Na_2_S, reduction of the azide group to the amino group (electron donating) occurs, activating the ICT process and turn-on fluorescence of the naphthalimide moiety. Probe 6 found applications for fluorescence imaging and ratiometric detection of H_2_S in live cells.

Han *et al.* engineered probe 7 integrated with triphenylphosphonium (mitochondria-targeting group), 2-fluoro-5-nitrobenzoic unit, and 1,8-naphthalimide fluorophore (well-known ICT fluorophore)^80^ for the detection of H_2_S_*n*_ (Fig. 8, Table 1).^81^ In the phosphate-buffered saline (PBS) solution, fluorescence titration of probe 7 with Na_2_S_2_ revealed a decrease in emission intensity at 485 nm. A new emission maximum at 550 nm emerged and increased (Stokes-shift = 109 nm). Probe 7 successfully demonstrated its application in imaging intracellular H_2_S_*n*_ with good selectivity and sensitivity.

SO_2_ is an essential endogenous signaling molecule that performs significant roles in many physiological processes. However, increased SO_2_ concentration is associated with severe lung cancer, nervous system diseases, and respiratory problems.^82–85^ Therefore, detecting SO_2_ and its derivatives in living systems becomes a high priority. Notably, a platform with fluorophores attached to aromatic heterocycles through C–C bonds was frequently used as a Michael addition receptor. The Michael-addition principle allows nucleophiles, such as bisulfite, to attack the C–C double bond.^86,87^ In this section, most probes for bisulfite detection follow the nucleophilic addition reaction mechanism.

**Fig. 9:**
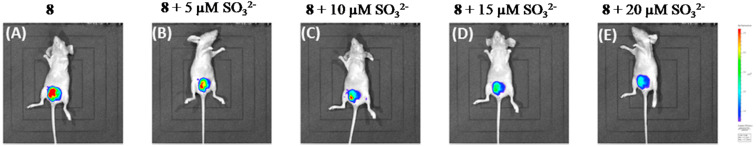
*In vivo* photos of nude mice (A) 8 (20 μM); (B–E) 8 (20 μM) + different concentrations of SO_3_^2−^ (5–20 μM). *λ*_ex_ = 530 nm, *λ*_em_ = 600–700 nm. Reprinted from ref. 88, copyright 2021 Elsevier.

Taking advantage of the merits of FRET-based systems such as a large Stokes-shift, Huang *et al.* developed near-infrared (NIR) fluorescent probe 8 by combining a coumarin carboxylic acid group with a piperazine substituted benzopyrylium salt (mitochondrion-targeting group) (Fig. 8, Table 1).^88^ In PBS solution, the fluorescence spectrum of probe 8 exhibited emission maxima at 635 nm and a large Stokes-shift (230 nm). The nucleophilic addition reaction of SO_3_^2−^ at the double bond of the benzopyrylium unit resulted in the interruption of the conjugated π-electron cloud, and the fluorescence at 635 nm decreased, accompanied by the simultaneous increase in the new emission peak at 455 nm. From the co-localization experiment, probe 8 efficiently targeted mitochondria (Pearson's co-localization coefficient = 0.84), and further investigations in HeLa cells and the nude mice experiment showed the application of probe 8 in biological systems for detecting SO_3_^2−^ (Fig. 9).

Liu *et al.* exploited probe 9, prepared by the condensation reaction between indolium or pyridinium and 3-formyl-9-methyl carbazole unit (Fig. 8).^89^ In the presence of HSO_3_^−^, probe 9 displayed ratiometric fluorescence change and the ratio of emission intensity (I_490_/I_590_) varied from 0.0383 to 3.8769 (101-fold enhancement). The *in vitro* imaging experiment proved the application of probe 9 for the quantification of SO_2_ derivatives in the mitochondria.

**Fig. 10:**
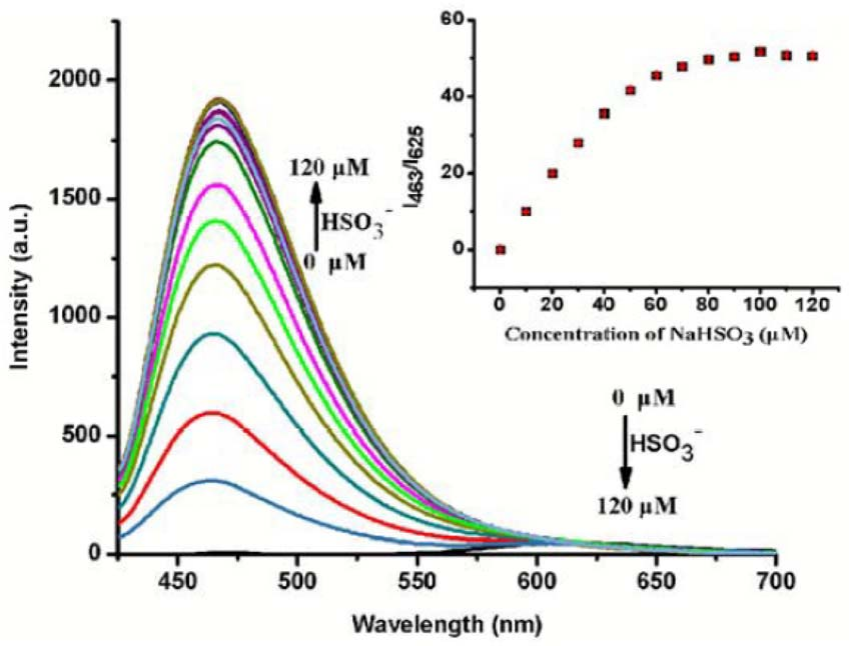
Fluorescence titration spectrum of probe 10 with HSO_3_^−^. Inset: plot of intensity ratio (I_463_/I_625_) *vs.* [HSO_3_^−^]. Reprinted from ref. 90, copyright 2016 Royal Society of Chemistry.

The same group presented another probe, probe 10, containing a carbazole and an alkyl sulfonated benzoindole (water soluble, mitochondria-targeting group) as the basic skeleton (Fig. 8, Table 1).^90^ In the presence of HSO_3_^−^, the emission spectrum of probe 10 revealed a blue shift of 162 nm, and the emission intensity ratio (I_463_/I_625_) was 56 (Fig. 10). The change in proton signals from 8.03–8.78 to 5.15 after the addition of HSO_3_^−^ in the ^1^H NMR spectrum, together with NOESY and COSY results, proved a 1,4-addition reaction-based detection mechanism. Unlike other reported probes for mitochondrial SO_2_ detection, probe 10 could monitor the mitochondrial SO_2_ level variation stimulated by carbonyl cyanide *m*-chlorophenyl hydrazone (CCCP) or by drugs.

Based on the 1,4-addition reaction between the polymethine chain of hemicyanine and SO_3_^2−^ or HSO_3_^−^, Wang *et al.* developed probe 11 to detect SO_2_ derivatives in aqueous medium and the mitochondria of living cells (Fig. 8, Table 1).^91^ Upon the addition of HSO_3_^−^, the fluorescence spectrum of probe 11 showed ratiometric fluorescence change and the plot of their ratio (I_467_/I_593_) *vs.* the concentration of HSO_3_^−^ was linear in the range of 1–9 μM. The sensing mechanism could be attributed to the interruption of the π-conjugation of probe 11 upon the addition of HSO_3_^−^ at the responsive site of probe 11, leading to a ratiometric change in the absorption and fluorescence spectrum. The fluorescence co-localization experiment revealed the unique distribution of probe 11 within the mitochondria of live MCF-cells (Pearson's correlation coefficients = 0.931, and Mander's overlap = 0.872). Furthermore, the applied probe 11 examined mitochondrial SO_2_ derivatives quantitatively through a fluorescence imaging experiment.

Wang *et al.* applied the strategy of combining two classical dyes to construct long-wavelength probe 12, prepared from benzopyrylium and chromenoquinoline dyes (Fig. 8, Table 1).^92^ The emission spectrum of the two-photon fluorescent probe showed a gradual decrease in red fluorescence at 613 nm (*λ*_ex_ = 580 nm) and an increase in blue fluorescence at 514 nm (*λ*_ex_ = 405 nm) upon the addition of HSO_3_^−^. The detection limit was 103 nM and 17 nM for the red and green channels, respectively. The nucleophilic addition reaction of HSO_3_^−^ at the C=C bond of probe 12 resulted in the interruption of π-conjugation/inhibition of the ICT process from the chromenoquinoline to the benzopyrylium group. Thus, a significant blue shift (99 nm) in the emission spectrum was observed. Furthermore, probe 12 displayed application for detecting SO_2_ derivatives in the solid state.

Wang *et al.* developed probe 13 based on pyrazoline (high quantum yield, cell permeability, and low cytotoxicity) and hemicyanine dyes (water soluble, mitochondrion-targeting group) (Fig. 8, Table 1).^93^ The fluorescence titration of probe 13 with SO_3_^2−^ revealed ratiometric fluorescence change, and the intensity ratio (I_480_/I_640_) changed from 0.45 to 445 (989 times). Intriguingly, probe 13 demonstrated an application for the ratiometric imaging of mitochondrial SO_2_ derivatives in living cells.

**Fig. 11:**
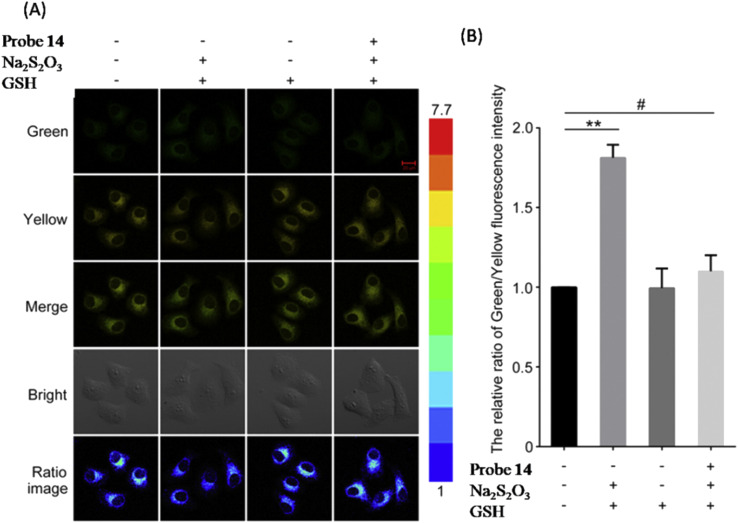
(A) Confocal microscopic images of HepG2 cells demonstrating the application of probe 14 for HSO_3_^−^ detection. (B) The relative intensity ratio (yellow/green) of columns 1, 2, 3 and 4 in (A). Reprinted from ref. 94, copyright 2017 Elsevier.

Keeping in mind Michael's addition principle and the FRET process, Wu *et al.* designed probe 14 based on the conjugated platform of dansyl, piperazine, and benzothiazolium, in which the benzothiazole moiety acts as a recognition unit and mitochondrion-targeting group (Fig. 8, Table 1).^94^ Upon the incremental addition of HSO_3_^−^, the fluorescence intensity ratio (I_540_/I_590_) of probe 14 showed a change from 0.3 to 1.5 (5-fold), and the limit of detection was 69 nM. Furthermore, probe 14 demonstrated a successful application for detecting HSO_3_^−^ in the mitochondria of living cells through fluorescence imaging (Fig. 11).

α,β-Unsaturated compounds are prone to a nucleophilic addition reaction by HSO_3_^−^. With this in mind, Xu *et al.* prepared probe 15 by the condensation of 1*H*-benzo[*e*]indolium (water soluble, high quantum yield, significant Stokes shift (>100 nm), and mitochondrion-targeting group) with carbazole-3-aldehyde (Fig. 8, Table 1).^95^ Upon the addition of HSO_3_^−^, the fluorescence spectrum of probe 15 underwent ratiometric fluorescence change with a decrease in the emission band at 588 nm and an increase in the new emission band at 462 nm, simultaneously. Interestingly, the cell staining experiment revealed probe 15 as cell-permeable and mitochondria-targetable, and it can monitor the intracellular SO_2_ derivatives in live cells (HeLa cells).

Zhang *et al.* constructed probe 16 composed of the coumarin-hemicyanine skeleton, in which the FRET process occurs from coumarin to hemicyanine and the ICT process occurs from the styryl to the indolium group (mitochondrion-targeting) (Fig. 8, Table 1).^96^ The nucleophilic approach of SO_3_^2−^ towards the double bond of hemicyanine resulted in disruption of π-conjugation and interruption in the ICT/FRET process. This dual emission signal of probe 16 changed in a see-saw manner with a significant emission shift (113 nm). The staining experiment revealed probe 16 as a specific mitochondrion-targeting probe and can detect endogenous SO_3_^2−^ in living cells such as HepG2 and L02 cells.

Zhao *et al.* developed probes 17 and 18 by using diethyl 2,2′-(phenylazanediyl)diacetate (electron-donor) and 1*H*-benzo[*e*]indolium (water soluble, high quantum yields, NIR emission property, and mitochondrion-targeting group) (Fig. 8, Table 1).^97^ In the presence of HSO_3_^−^/SO_3_^2−^, probe 17 and 18 displayed ratiometric fluorescence change, blue-shift in emission (over 100 nm), and high selectivity and sensitivity (LOD = 0.20 μM and 0.11 μM for probes 17 and 18, respectively). The sensing mechanism for both probes was a nucleophilic addition reaction. However, the path of the sensing mechanism was different. Interestingly, both the probes displayed the capability of quantifiable tracking and fluorescence imaging of HSO_3_^−^/SO_3_^2−^ in the mitochondria of living cells.

Zheng *et al.* constructed probe 19 by the condensation reaction between trimethylbenzoindolium (mitochondrion-targeting moiety) and *p*-diphthalaldehyde (Fig. 8, Table 1).^98^ The fluorescence response of probe 19 toward HSO_3_^−^ was initially ratiometric (at low concentrations) and then turn-on emission (at high concentrations). The sensing mechanism was a twice nucleophilic addition reaction. The cell imaging revealed that the probe 19 could specifically detect HSO_3_^−^ in the mitochondria of living cells (HepG2 cells).

### 4.3 Fluoride detection in mitochondria

The fluoride ion is one of the most significant anions crucial to many biological and medicinal processes. According to literature reports, a high fluoride ion concentration can damage mitochondria through oxidative stress and reduce the mitochondrial respiratory chain's efficiency, resulting in mitochondrial malfunction and the development of neurodegenerative disorders.^99^ As a result, it is essential and beneficial to monitor fluoride ions in mitochondria.

Shen *et al.* applied ICT-modulated strategies for constructing probe 20 based on a diethylaminocoumarin derivative (a well-known ICT fluorophore) in which a pyridinium salt behaves as a mitochondrion-targeting group (Fig. 8, Table 1).^100^ Initially, probe 20 exhibited red fluorescence at 639 nm (*λ*_ex_ = 490 nm) attributed to the ICT process between the pyridinium cation and 7-diethylamino-coumarin unit. Upon the addition of F^−^, a new emission band at 539 nm emerged and increased at the expense of a decrease in emission at 639 nm. The sensing mechanism was F^−^-induced cleavage of the Si–O bond between the phenyl and triisopropylsilyl groups. Furthermore, the applied probe 20 successfully examined mitochondrial F^−^ in living cells.

### 4.4 Palladium ion (Pd^2+^) detection in mitochondria

Due to its high stability and reliability characteristics, palladium has been extensively employed in various applications, including catalysis, synthesis of organic compounds, medicine, dental crowns, fuel cells, and electronics production. However, palladium ion enrichment in living organisms can cause severe health-related diseases.^101,102^ Therefore, developing an efficient tool for palladium ion detection in a biological system is necessary.

Wang *et al.* developed probe 21 to detect Pd^2+^ in living cells based on the FRET process and rhodamine ring-opening mechanisms (Fig. 8, Table 1).^29^ In PBS buffer solution, free probe 21 exhibited emission maxima at 472 nm (*λ*_ex_ = 400 nm). Upon adding Pb^2+^, a decrease in emission at 472 nm and an increase in the new emission band at 594 nm were observed. Moreover, the fluorescence imaging experiment revealed the application of probe 21 for the ratiometric visualization of Pd^2+^ in the mitochondria of living cells.

### 4.5 Challenges

Most organelle-targeting methods discussed above focus on lipophilic cationic fluorescent probes that selectively target mitochondria. However, problems with cationic probes, such as the effect on membrane potential and cellular toxicity, are yet to be overcome. Furthermore, to explain the probe's potential to target mitochondria, mostly co-localization experiments have been performed using commercially available mitochondria-specific dyes such as MitoTracker Green FM, MitoTracker Red, and MitoTracker Orange. However, there can be several other contributing factors and principles behind the probe's potential to target mitochondria, and these factors need a clear discussion.

In addition, mitochondria contain hundreds of biomolecules, such as anions, cations, enzymes, mitochondrial DNA, RNA, lipids, and so forth. Using fluorescent probes, it is still challenging to selectively label bioactive compounds at low concentrations (often nanomolar levels). No doubt, ratiometric fluorescent probes hold promise for removing various background interferences. However, nonetheless, just a few mitochondrion-targeting ratiometric fluorescent probes have been developed to date.

## 5 Lysosome-targeting ratiometric fluorescent probes

Lysosomes, an essential subcellular organelle, which can function as a digestive compartment in eukaryotic cells and include a variety of enzymes and proteins, are critical regulators in metabolic processes under acidic pH circumstances.^103^ As key indicators of lysosome function and oxidative stress, reactive oxygen species (ROS) such as HClO and H_2_O_2_ and reactive sulphur species (RSS) such as H_2_S have been the focus of numerous probe designs. Since the lysosome lumen is acidic, developing probes that can detect HOCl in an acidic medium is challenging.^4,30^

### 5.1 Reactive oxygen species (ROS) detection in the lysosome

H_2_O_2_ is a significant reactive oxygen species (ROS) with distinct destructive oxidation characteristics. Lysosomes can produce hydrogen peroxide (H_2_O_2_) to combat pathogens.^104–106^ Therefore, it's crucial to create a reliable method for measuring H_2_O_2_ in inflammatory tissues to assess the physiological and pathological link between lysosomal H_2_O_2_ and inflammation.

**Fig. 12:**
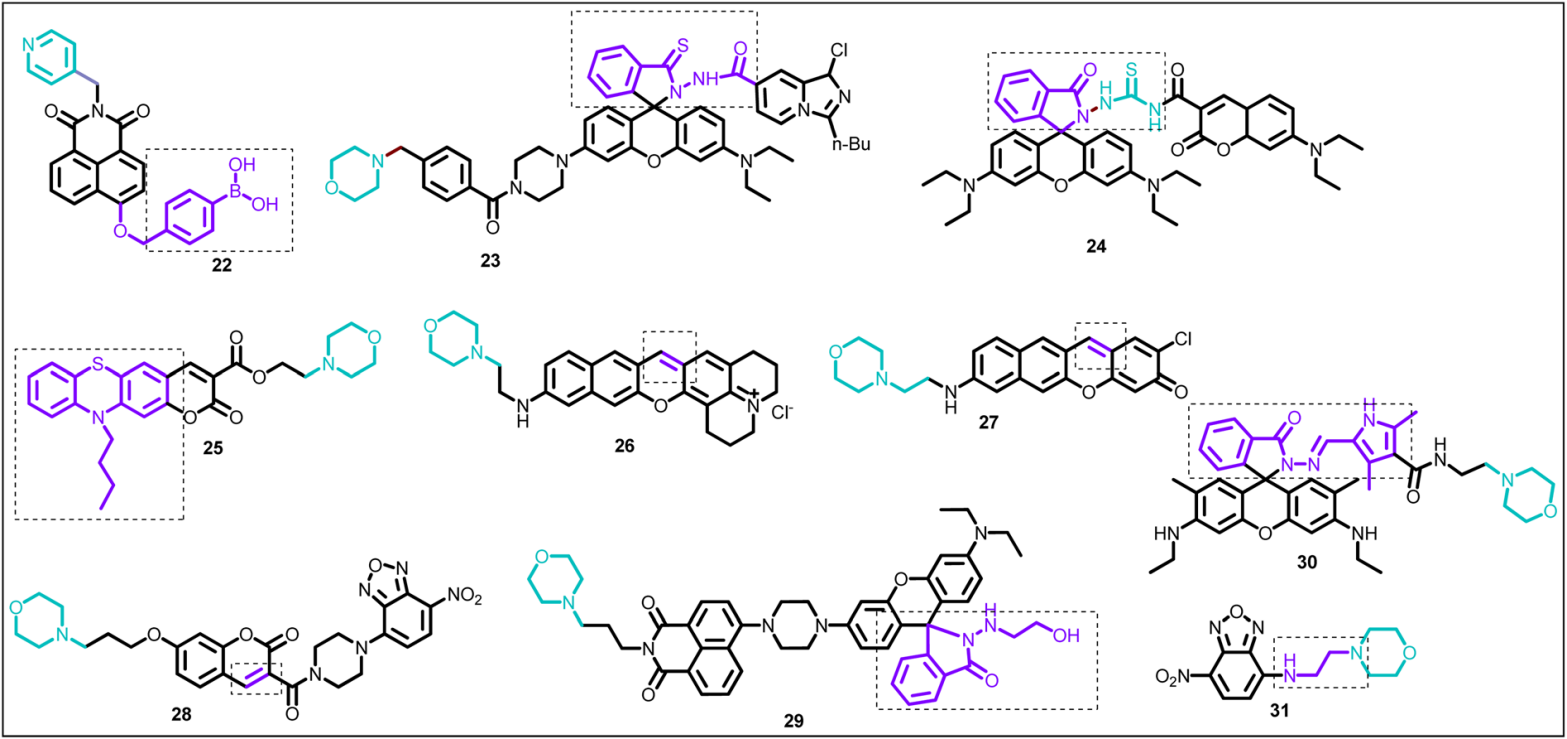
Chemical structure of lysosome-targeting ratiometric fluorescent probes (22–31), (brown color: lysosome-targeting unit; purple color with dotted box: response site).

Inspired by the excellent optical properties of naphthalimide derivatives (donor–π-acceptor structured), Zhou *et al.* constructed probe 22 based on naphthalimide, benzylboric acid (H_2_O_2_ responsive group), and pyridine group (lysosome-targeting group) for monitoring H_2_O_2_ in living tissue and in inflamed tissue (Fig. 12, Table 1).^37^ In the presence of H_2_O_2_, probe 22 displayed ratiometric fluorescence change, assigned to H_2_O_2_ mediated removal of the boric acid group from probe 22 and ICT effect. The fluorescence color of the solution changed from bright blue to light yellow. Furthermore, tissue imaging experiments using a confocal microscope demonstrated a potential application for H_2_O_2_ detection in inflamed tissues.

TBET-based probes offer several advantages, such as high energy transfer efficiencies, improved imaging resolution, and a large Stokes-shift.^107,108^ With this in mind, Shen *et al.* synthesized probe 23 based on the imidazo[1,5-*a*]pyridine moiety (donor) and rhodamine moiety (acceptor) (Fig. 12, Table 1).^15^ The free probe 23 exhibited emission at 462 nm attributed to the emission of the imidazo[1,5-*a*]pyridine fluorophore. Upon the gradual addition of aliquots of HOCl, the emission intensity at 462 nm almost remained constant, while a new emission maximum at 589 nm emerged and increased, assigned to the rhodamine moiety. The sensing mechanism was the change of the rhodamine spiro form to the ring-open state in the presence of HOCl and the TBET process between the rhodamine unit and imidazo[1,5-*a*]pyridine. Applied probe 23 successfully monitored the HOCl changes in the lysosomes.

Using a similar mechanism to the one described above, which converts the rhodamine spiro-form into the ring-open form when HOCl is present, Yuan *et al.* provided the coumarin and rhodamine based FRET platform 24 for HOCl detection in living cells (Fig. 12, Table 1).^14^ Upon excitation at 410 nm, probe 24 displayed emission at 480 nm (which belonged to the coumarin moiety). However, in the presence of HOCl, the emission spectrum of probe 24 showed a decrease in intensity at 480 nm and an increase in new maxima at 580 nm (which belonged to rhodamine), assigned to a ring-opening and FRET (FRET efficiency = 93.75%) based detecting mechanism.

**Fig. 13:**
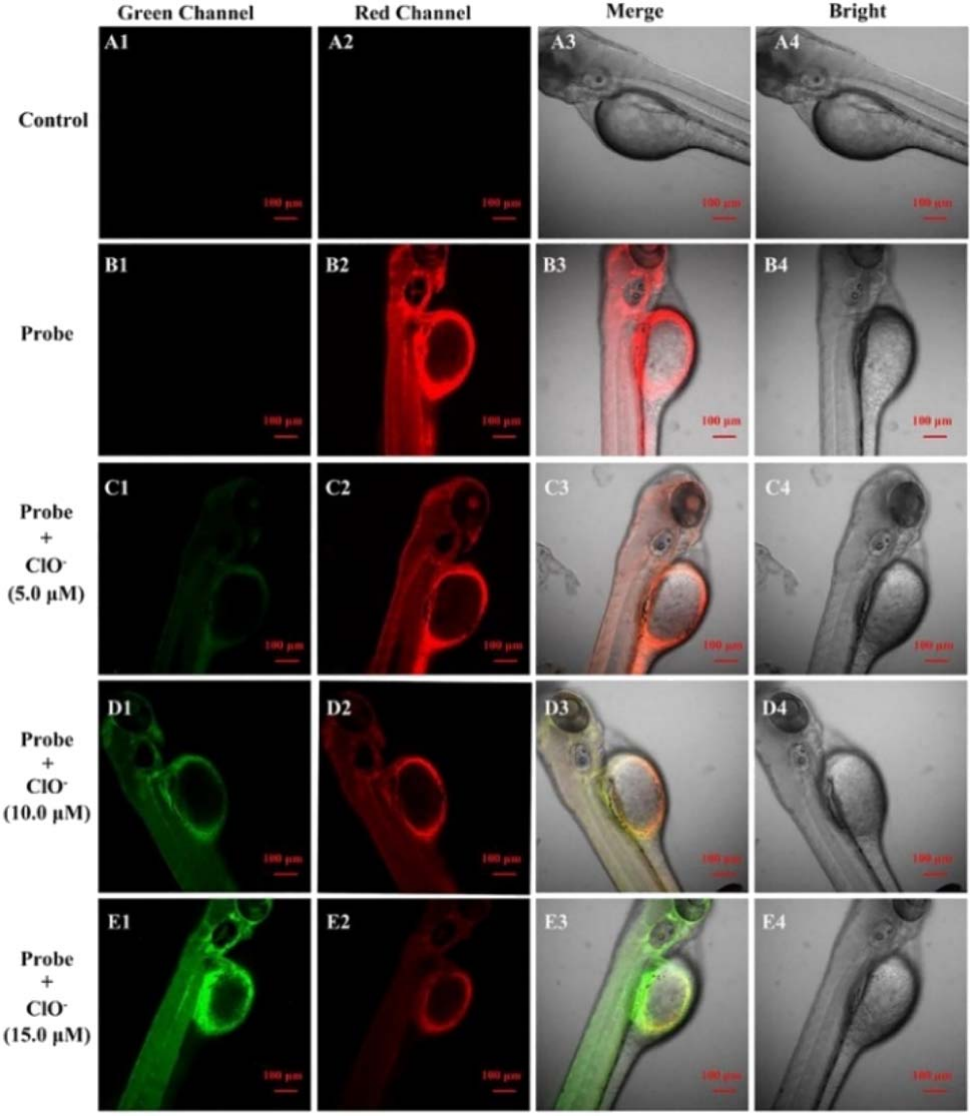
Fluorescence images of zebrafish. The control group (A1–A4). Zebrafish co-cultured with probe 25 (10 μM) (B1–B4), followed by incubation with ClO^−^ 5 μM (C1–C4), 10 μM ClO^−^ (D1–D4) and 15 μM ClO^−^ (E1–E4). Reprinted from ref. 109, copyright 2021 Elsevier.

Liu *et al.* constructed probe 25 from phenothiazine coumarin and a morpholine unit for hypochlorite detection (Fig. 12, Table 1).^109^ The fluorescence titration of probe 25 with ClO^−^ showed a blue shift in emission from 610 to 535 nm. The sensing mechanism was inhibition of the ICT process due to the oxidation of the phenothiazine moiety. Furthermore, the fluorescence imaging experiment demonstrated the application of probe 25 for detecting ClO^−^ in living cells (RAW264.7 cells) and zebrafish (Fig. 13).

### 5.2 Reactive sulfur species (RSS) detection in lysosome

The endogenous oxidation of hydrogen sulfide or sulfur-containing amino acids produces bisulfite, which remains in equilibrium with sulfur dioxide and sulfite in aqueous media.^110–113^ As a result, the discovery of bisulfite in lysosomes is of great interest.

Tamima *et al.* pioneered probe 26 by introducing a morpholine moiety (targeting group) to the benzopyronin dye (Fig. 12, Table 1).^114^ After adding bisulfite, the absorption and fluorescence spectrum of probe 26 displayed complete peak separation from 613 nm to 426 nm (spectral shift = 187 nm) and from 704 nm to 512 nm (spectral shift = 192 nm), respectively. The sensing mechanism was the 1,6-conjugate addition reaction. Probe 26 specifically targeted lysosomes and displayed application for monitoring intracellular bisulfite levels.

Cysteine, homocysteine, hydrogen sulfide, and glutathione are examples of cellular thiols frequently studied employing lysosome-targeting probes.^115^ These biothiols are significant indications of lysosomal function because they are produced by lysosomal proteolysis.^116^

Tamima *et al.* devoted benzo[*b*]xanthene-derived probe 27 for the two-photon ratiometric fluorescence-based detection of cysteine (Fig. 12, Table 1).^117^ The detecting mechanism of cysteine was a 1,6-conjugate addition reaction to the benzoxanthene core, resulting in the formation of a cysteine adduct that emitted red. However, in the presence of hydrogen peroxide, the 1-cysteine adduct was reverted to 27. Furthermore, probe 27 demonstrated practical application to quantify cysteine levels in biological samples (human blood plasma).

Zhang *et al.* applied a cleavable FRET-based strategy for designing a lysosome-targeting probe 28, based on a coumarin–NBD (nitrobenzofurazan) cassette (Fig. 12, Table 1).^118^ Free probe 28 showed two emission maxima at 415 nm and 560 nm, belonging to the coumarin and NBD groups. Upon treatment with H_2_S, probe 28 underwent ratiometric fluorescence change, the solution's emission color changed from yellow to blue, and the intensity ratio increased from 0.22 to 67.7 (300-fold approx.). Furthermore, probe 28 preferentially targeted lysosomes and displayed potential application for detecting H_2_S in lysosome.

### 5.3 Cu^2+^ detection in the lysosome

Many essential physiological processes involve the role of copper. Copper plays important roles in a variety of fundamental physiological processes.^119^ At the organelle level, improper copper homeostasis can cause several severe illnesses.^120–122^ As a result, it is still essential to monitor the concentration of copper levels in cells, particularly in lysosomes.

Liu *et al.* applied FRET-based strategies for constructing ratiometric probe 29 (Fig. 12, Table 1).^123^ The probe 29 displayed high selectivity, sensitivity (detection limit = 1.45 nM), and ratiometric fluorescence response towards Cu^2+^. From fluorescence imaging experiments, live cells (L929 cells) incubated with probe 29 showed blue and weak green fluorescence. However, cells pre-treated with Cu^2+^ and then incubated with probe 29 showed solid green and diminished blue fluorescence. In addition, probe 29 was applied for fluorescence imaging of Cu^2+^ in the lysosome of living cells.

Inspired by Czarnik's report on Cu^2+^-induced ring opening of rhodamine,^124^ Wu *et al.* synthesized probe 30 for Cu^2+^ detection (Fig. 12, Table 1).^125^ In CH_3_CN–H_2_O (8 : 2) solution, probe 30 behaved as a selective and sensitive probe for Cu^2+^ over other tested ions. The sensing mechanism was Cu^2+^-induced ring opening and the FRET process from pyrrole to rhodamine.

### 5.4 Hg^2+^ detection in the lysosome

Mercury is a hazardous and pervasive heavy metal that can result in numerous serious health issues, including renal failure, damage to the central nervous system, *etc.*^126^ Therefore, it is crucial to have an effective method for detecting mercury ions.

Zhang *et al.* developed an NBD-based probe 31 by introducing a morpholine moiety to the NBD fluorophore for Hg^2+^ detection (Fig. 12, Table 1).^127^ In the presence of Hg^2+^, the absorption spectrum of probe 31 showed a red shift of the peak from 470 nm to 494 nm, and the color detected by the naked eye changed from light yellow to red. Under similar conditions, the fluorescence spectrum also revealed a red shift of emission maxima from 535 nm to 595 nm. Herein, the morpholine unit played dual roles as a ligand for Hg^2+^ and lysosome-targeting group. The sensing mechanism was assigned to the inhibition of the PET process from the nitrogen atom of morpholine to the NBD fluorophore upon coordination with Hg^2+^.

### 5.5 Challenges

Lysosome-based research has advanced significantly in recent years. Nevertheless, there are still several challenges for these probes. The synthetic probe cannot differentiate between autolysosomes, autophagosomes, endosomes, and other acidic compartments. These probes are harmful to live cells and inappropriate for long-term detection because they make lysosome alkaline. As a result, lysosome-specific probes devoid of the alkalization effect are necessary. Most of the developed probes have emissions in the visible region, thus, cannot be used for deep-tissue imaging due to the poor penetration power.

## 6 Endoplasmic reticulum-targeting ratiometric fluorescent probes

The most prominent organelle in a cell, the endoplasmic reticulum (ER), is crucial for protein synthesis, folding, distribution, and calcium ion storage. Literature reports revealed that ER stress, which is linked to significant diseases, can cause autophagy and even cell death. Therefore, studying ER is an exciting field of research.^128,129^

### 6.1 Reactive oxygen species (ROS) detection in endoplasmic reticulum

H_2_O_2_, an important reactive oxygen species, plays a crucial role in various pathological and physiological processes. During ER stress, the increased concentration of H_2_O_2_ can damage cellular proteins and may result in cancer, metabolic diseases, cardiovascular diseases, neurodegenerative diseases, *etc.*^130,131^ Therefore, developing efficient analytical methods for H_2_O_2_ in ER is crucial.

**Fig. 14:**
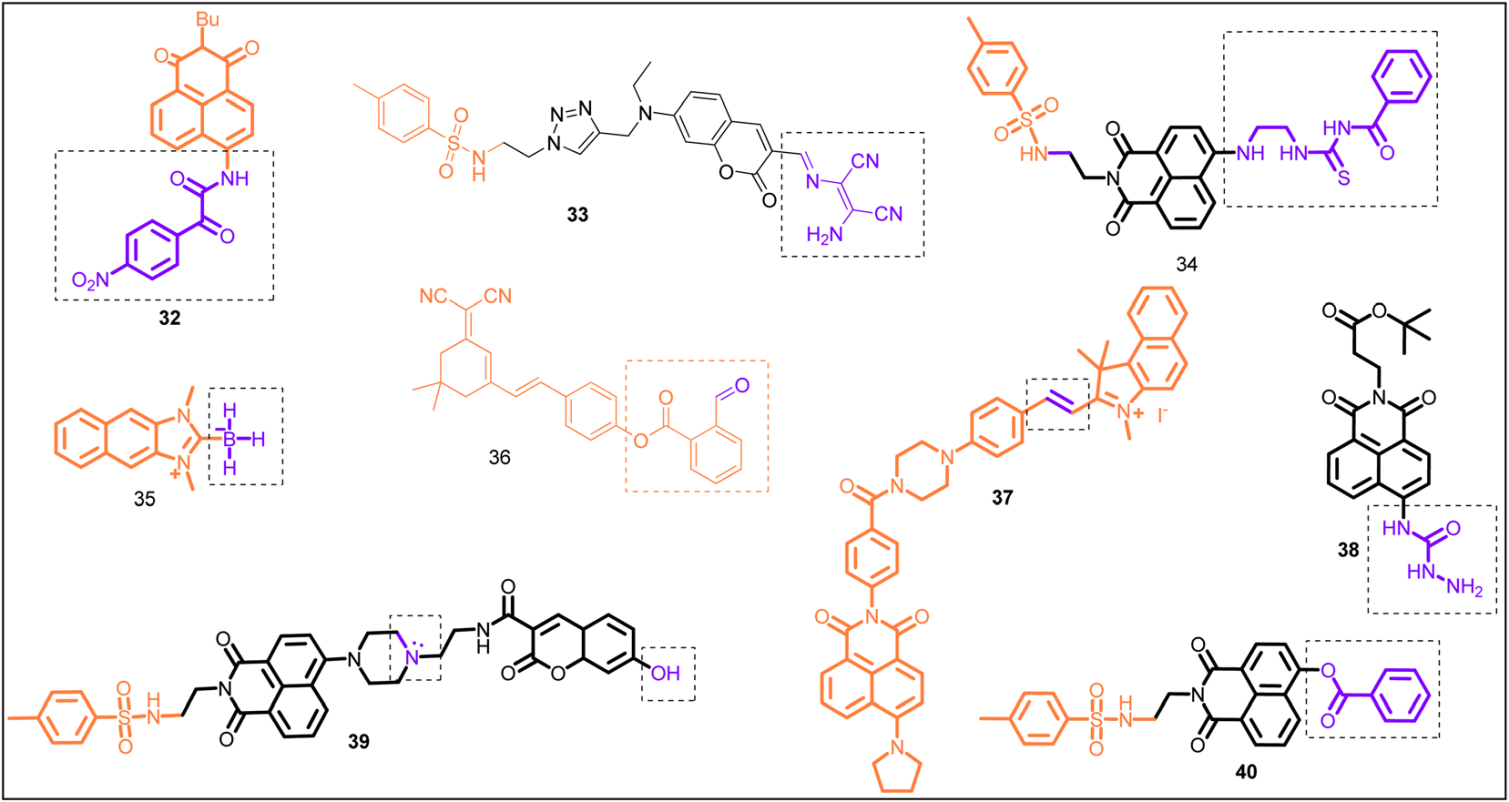
Chemical structure of endoplasmic reticulum-targeting ratiometric fluorescent probe (32–40), (orange color: ER-targeting unit; purple color with dotted box: response site).

In the past, researchers developed various probes for detecting exogenous H_2_O_2_ in ER. However, quantitative detection of endogenous H_2_O_2_ remains challenging. To solve this issue, Gao *et al.* synthesized probe 32 based on α-ketoamide and naphthalimide groups (Fig. 14, Table 1).^131^ Upon adding H_2_O_2_, the fluorescence spectrum of probe 32 revealed ratiometric fluorescence change, attributed to the reaction between H_2_O_2_ and α-ketoamide and subsequent hydrolysis of amido linkage. The limit of detection was 38 nM for H_2_O_2_. Furthermore, applied probe 32 found application for quantitative measurement of endogenous H_2_O_2_ in ER of living cells (HeLa cells), both under normal conditions (0.692 μM H_2_O_2_) and under ER stress (1.26 μM H_2_O_2_).

In the past, the diaminomalenonitrile-based Schiff base has been used by several researchers for developing ClO^−^ specific sensors.^132–134^ On the other hand, the sulfonamide group has been known to target ER.^135^ Taken together, Hou *et al.* constructed biosensor 33 for ClO^−^ detection in ER (Fig. 14, Table 1).^39^ Upon the addition of ClO^−^, the fluorescence spectrum of biosensor 33 displayed a blue shift in emission wavelength from 554 nm to 480 nm, attributed to the reaction-based sensing mechanism. The plot of emission intensity ratio (I_480_/I_554_) *vs.* ClO^−^ concentration was linear between 0 and 120 μM concentrations. The detection limit was 0.59 μM for ClO^−^. The applied biosensor 33 examined exo/endogenous ClO^−^ in the ER of living cells.

Inspired by the excellent optical properties and ICT process of 4-aminonaphthalimide moiety,^136^ Ma *et al.* constructed biosensor 34 using a 4-aminonaphthalimide moiety (fluorescent group), (2-aminoethyl) thiourea unit (HClO recognition site), and *p*-toluenesulfonamide unit (ER-targeting group) (Fig. 14, Table 1).^41^ In ethanol–H_2_O (1 : 1, v/v) solution, free sensor 34 showed emission at 533 nm (green emission), attributed to the ICT process. After adding HOCl, biosensor 34 revealed ratiometric fluorescence change with blue-shift (49 nm) of the emission maxima from 533 nm to 484 nm, which was assigned to inhibition of the ICT process. Furthermore, biosensor 34 demonstrated an application for HOCl detection in the ER of PC-12 cells.

In the past, aryl boronic acid has been widely utilized in designing fluorescent sensors for biological species,^137^ such as peroxynitrite (ONOO^−^),^138^ hypochlorite (OCl^−^)^139^ and hydrogen peroxide.^140,141^ Pak *et al.* designed NHC-borane-based fluorescent sensor 35, constructed from a naphthoimidazolium precursor (HOCl responsive unit) for specific detection of HOCl over other ROS (Fig. 14, Table 1).^18^ Sensor 35 followed an electrophilic oxidation mechanism that involved B–H bond cleavage. Sensor 35 successfully targeted ER and demonstrated an application for monitoring HOCl in living cells (Raw 264.7 cells) and rat hippocampal slices by a two-photon fluorescence microscopy experiment.

### 6.2 Reactive sulfur species (RSS) detection in endoplasmic reticulum

Taking into account the essential role of H_2_S during ER stress and ER functions,^142,143^ Shu *et al.* pioneered ratiometric NIR-fluorescent biosensor 36 based on dicyano-isophorone (NIR emission, large Stokes-shift) and an *O*-carboxybenzaldehyde unit (H_2_S recognition site) for H_2_S detection in ER (Fig. 14, Table 1).^144^ Sensor 36 showed fluorescence emission at 560 nm due to the ICT effect. Upon the gradual addition of H_2_S, a new emission peak at 650 nm appeared and increased with the simultaneous decrease in *λ*_em_ at 560 nm. The sensing mechanism was a nucleophilic addition reaction. Furthermore, sensor 36 demonstrated an application for monitoring H_2_S in living cells (HeLa cells) and zebrafish. Interestingly, sensor 36 successfully detected endogenous H_2_S produced during ER stress incited by Tunicamycin.

Nowadays, physiological functions of SO_2_ and its derivatives are gaining increasing attention. According to literature reports, SO_2_ is vital in cardiovascular processes^145^ and can regulate hippocampal neuron apoptosis.^146^ Thus, SO_2_ detection is essential. Li *et al.* developed a FRET-based platform 37, constructed from benzoindole-based hemicyanine (acceptor) and naphthalimide derivatives (donor), for the detection of SO_2_ derivatives in ER (Fig. 14, Table 1).^147^ Sensor 37 exhibited emission maxima in buffer solution at 610 nm (*λ*_ex_ = 440 nm), attributed to the FRET process. The sensing mechanism was a nucleophilic addition reaction and inhibition of the FRET process. Furthermore, sensor 37 successfully targeted ER and demonstrated an application for imaging exogenous and endogenous SO_2_ derivatives in living cells.

### 6.3 Cu^2+^ detection in endoplasmic reticulum

Mitochondria and lysosomes implicate the cellular homeostasis of copper.^148^ Furthermore, lysosomes in damaged tissue contained high concentrations of copper ions.^149^ However, it is unclear whether or not the copper ions in these organelles are the cause of the ER's harmful actions. Thus, to understand copper-related diseases, a reliable technique for imaging copper at the level of organelles is crucial.

Park *et al.* presented a naphthalimide and hydrazide based biosensor 38 for copper ion detection in living cells (Fig. 14, Table 1).^150^ Upon the addition of copper ion to the solution of sensor 38 in HEPES buffer, the emission maxima were red-shifted (380 nm to 440 nm), and solution color changed from blue to yellowish-green. The detecting mechanism was assigned to the copper-mediated hydrolytic reaction of sensor 38, forming aminonaphthalimide. Additionally, the biocompatible sensor 38 specifically targeted the ER of living cells and displayed potential for qualitative and quantitative detection of Cu^+^/Cu^2+^ under physiological conditions.

### 6.4 Monitoring pH in endoplasmic reticulum

The physiological functions of the ER, such as targeting during secretion, protein sorting, and retrieving resident chaperones, are regulated by the pH of the ER, which serves as a crucial parameter.^151^ The ER pH is the same as that of the cytoplasm under normal physiological conditions.^152^ Recent research has shown that ER stress potently stimulates autophagy, strongly linked to many diseases (such as cancer, infectious disorders, and neurodegeneration), causing the ER pH to drop.^153,154^ Therefore, it is crucial to quantitatively evaluate the pH change in ER to explain the biological functions of ER fully.

**Fig. 15:**
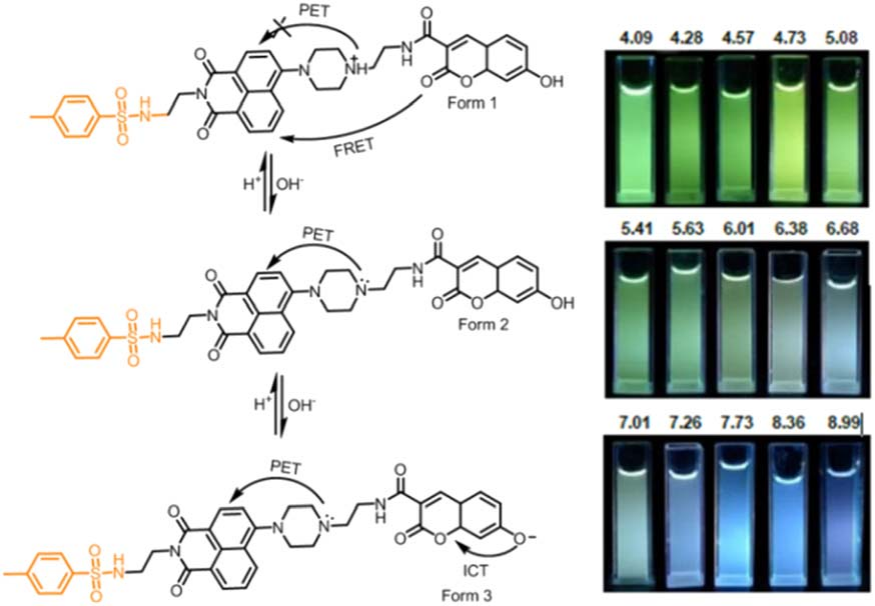
(A) The sensing mechanism of probe 39 to pH. (B) Fluorescence images of probe 39 at different pH under illumination at 365 nm. Reprinted from ref. 40, copyright 2019 Royal Society of Chemistry.

Fluorescent probes with dual responsive sites have shown improved sensitivity to pH and enhanced emission wavelength.^155^ With this in mind, Dong *et al.* pioneered probe 39 based on the naphthalimide–coumarin platform and employed hydroxyl and morpholine groups as the pH-responsive sites (Fig. 14, Table 1).^40^ On excitation at 405 nm, the fluorescence spectrum of probe 39 showed ratiometric change with a single emission band at 527 nm (acidic pH, 4.09–5.08) and two emission maxima at 446 nm and 527 nm between pH 5.08 and 7.73, and further single emission maxima at 446 nm at pH 7.73 (basic pH). The sensing mechanism was assigned to the FRET–PET–ICT process (Fig. 15). Furthermore, due to the presence of the *p*-toluenesulfonamide group (ER-targeting group), probe 39 successfully targeted ER. It demonstrated an application for quantitatively detecting the pH changes in the dexamethanose-treated cell and during ER stress.

### 6.5 Carboxylesterase 2 (CES2) detection in endoplasmic reticulum

**Fig. 16:**
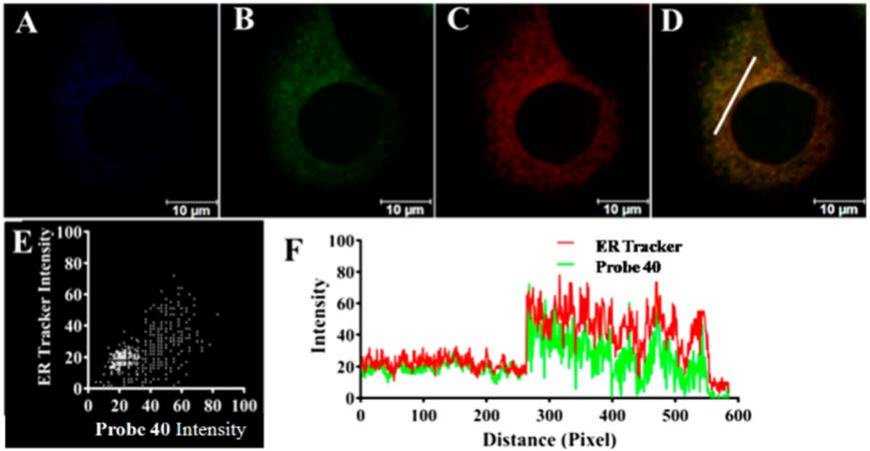
Fluorescence images of probe 40 in HepG2 at the (A) blue channel (425–475 nm); (B) at the green channel (535–585 nm); (C) ER-Tracker (red); (D) merged image (green channel and ER-Tracker channel (red)); (E) intensity scatter plot; and (F) intensity plot for the region of interest (white line in panel D). Reprinted from ref. 158, copyright 2019 American Chemical Society.

Carboxylesterase 2 (CES2) has critical roles in ER, such as metabolic, drug detoxification, molecular target for the prodrug design, and ER stress-associated diseases.^156,157^ Tian *et al.* developed ratiometric fluorescent probe 40 based on *p*-toluenesulfonamide (ER-targeting group) for CES2 detection (Fig. 14, Table 1).^158^ Among various hydrolases, only CES2 catalyzed the hydrolysis of probe 40. Probe 40 was applied for imaging CES2 in living cells (HepG2 cells) and tumor tissue (Fig. 16). Notably, Probe 40 revealed a significant decrease in CES2 activity under ER stress and drug (acetaminophen (APAP))-induced liver injury model.

### 6.6 Challenges

Over the past few years, scientists engineered several ER-targeting fluorescent probes for the selective detection of various substances like NO, H_2_S, H_2_O_2_, and HOCl. However, the mechanism behind ER selectivity still needs to be clarified. Many biological species present in ER still need to be detected due to a lack of efficient probes. In addition, most of the probes discussed above emit visible light. Thus, their *in vivo* applicability is restricted.

## 7 Golgi apparatus-targeting ratiometric fluorescent probes

The Golgi apparatus is an organelle with a phospholipid membrane composed of cisterna. It transforms proteins from the rough ER, then separates them into vesicles for transport to other cell regions. According to recent findings, CO is crucial for the Golgi apparatus.^159–161^ Thus, to study in depth various functions of the Golgi apparatus, it is essential to develop efficient Golgi apparatus-targeting probes.

### 7.1 Reactive oxygen species (ROS) detection in Golgi apparatus

**Fig. 17:**
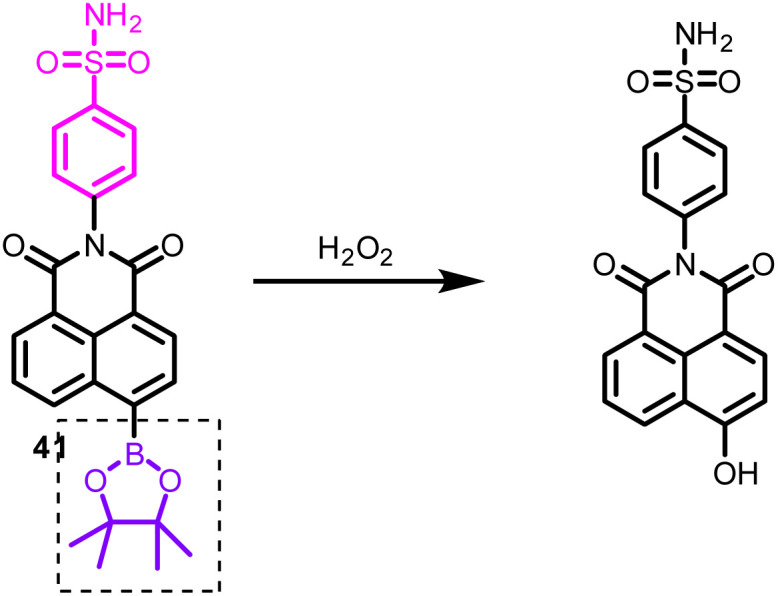
Reaction-based detection mechanism of probe 41 for H_2_O_2_.

Golgi oxidative stress is closely linked to the occurrence and progression of hypertension, and the concentration of hydrogen peroxide (H_2_O_2_) plays a critical role in this process. To address this issue, Wang *et al.* developed a two-photon fluorescent probe 41, which targets the Golgi apparatus with the aid of a phenylsulfonamide group. The response mechanism was a reaction-based process (Fig. 17).^162^ Upon reacting with H_2_O_2_, the boric acid ester is transformed into a hydroxyl group that donates electrons, thereby promoting the push–pull electron effect of the naphthalimide-conjugated system. This leads to the production of strong fluorescence emission. The probe 41 enables *in situ* H_2_O_2_ ratiometric imaging in living systems and provides a highly effective means to monitor Golgi oxidative stress. The probe 41 was able to identify the generation of H_2_O_2_ during Golgi oxidative stress and demonstrated increased levels of Golgi H_2_O_2_ in the kidneys of hypertensive mice.

### 7.2 Reactive nitrogen species (RNS) detection in Golgi apparatus

**Fig. 18:**
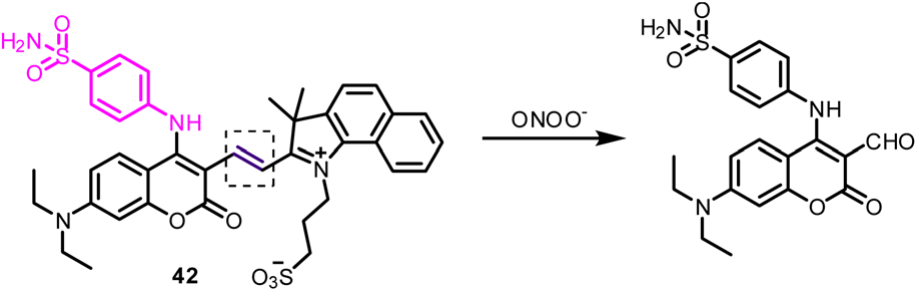
Reaction-based detection mechanism of probe 42 for ONOO^−^.

The detection of peroxynitrite (ONOO^−^) is essential for the study and treatment of drug-induced liver injury (DILI) associated with oxidative stress. Targeting the Golgi apparatus has emerged as a new approach for DILI research and treatment. Feng *et al.* developed a new probe, 42, by conjugating a sulfanilamide moiety to a coumarin–hemi-cyanine conjugated system (Fig. 18).^163^ The probe displayed high sensitivity, selectivity, and low cytotoxicity, and showed a rapid ratiometric fluorescence response to ONOO^−^. Unexpectedly, probe 42 also displays unique targeting properties in living cells, with the ability to label the cell membrane first and then the Golgi. Imaging experiments with probe 42 showed it to be effective in monitoring ONOO^−^ under Golgi oxidative stress and in DILI using mice models.

### 7.3 CO monitoring in Golgi apparatus

**Fig. 19:**
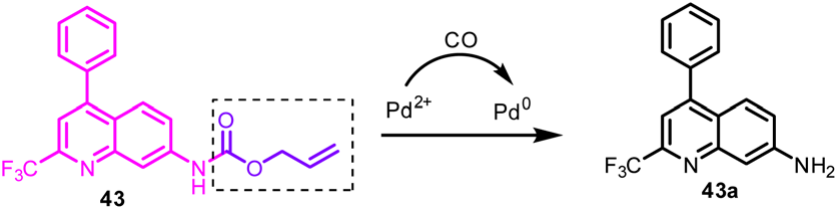
Sensing mechanism of probe 43 for CO detection based on Tsuji–Trost reaction.

**Fig. 20:**
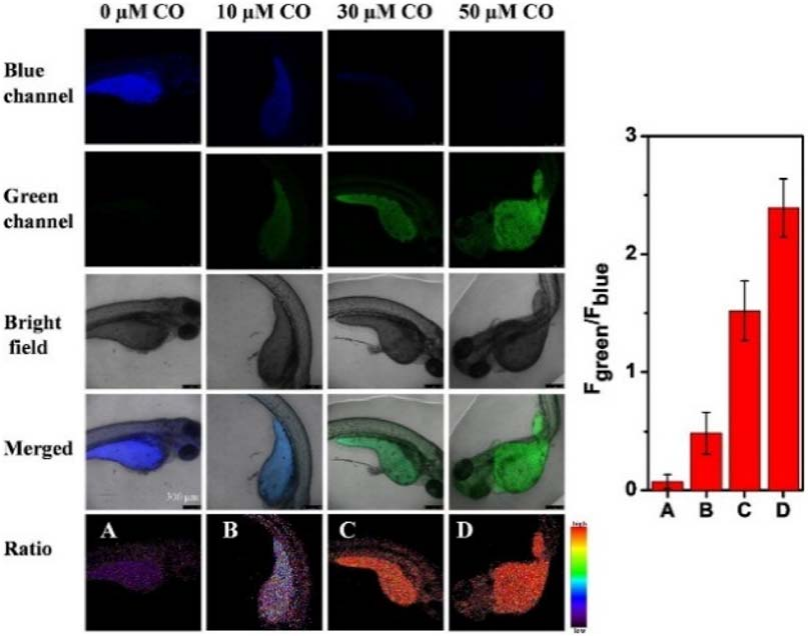
CO fluorescence imaging in zebrafish with probe 43. The right figure exhibits the fluorescence intensity ratio value (green/blue). Reprinted from ref. 164, copyright 2021 Elsevier.

Inspired by the recent reports on the fluorescent probe for CO detection based on the Tsuji–Trost reaction and to understand in depth the role of CO in subcellular organelles, Zheng *et al.* constructed probe 43 (Fig. 19, Table 1). On excitation at 360 nm, probe 43 exhibited emission at 425 nm.^164^ Upon the addition of CORM-3 (CO donor), probe 43 showed a significant 95 nm red shift with a decrease and increased emission maxima at 425 nm and 560 nm, respectively. The detecting mechanism was attributed to the cleavage of the allycarbamate group of probe 43 to form 43a mediated by a Pd^0^ Tsuji–Trost reaction. Furthermore, probe 43 found application for CO imaging in cells and zebrafish and to visualize CO levels during cellular oxidative stress stimulated by lipopolysaccharide (Fig. 20).

### 7.4 Glutathione (GSH) detection in Golgi apparatus

**Fig. 21:**
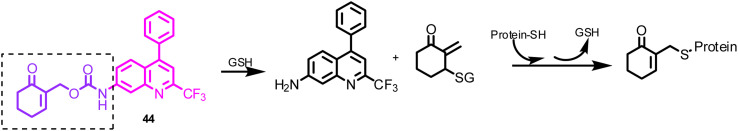
Reaction-based detection mechanism of probe 44 for GSH.

The high expression of antioxidants like glutathione in cancer cells helps them withstand oxidative stress within the Golgi apparatus. Thus, monitoring changes in glutathione concentration within the Golgi could serve as an effective way to track the occurrence and progression of tumor cells. Rong *et al.* created a Golgi-targeting probe 44 that could detect GSH with high accuracy.^165^ The fluorescence titration experiment of probe 44 with GSH revealed a decrease in intensity at 425 nm and emergence of a new peak at 510 nm. The detection limit was 0.49 μM for GSH. The response mechanism was a reaction-based process (Fig. 21). Furthermore, the Golgi stress response experiment revealed the ability of probe 44 for *in situ* endogenous GSH detection in the Golgi apparatus during oxidative stress.

### 7.5 Challenges

The Golgi apparatus-targeting ratiometric fluorescent probes are rare in the literature. Furthermore, the targeting mechanism is not clear.

## 8 Nucleus-targeting ratiometric fluorescent probes

For both cancer treatment and genetic engineering, the cell nucleus has been a primary target since it stores the genetic material that is protected by the nuclear envelope, which is made up of two lipid bilayer membranes.^166^

### 8.1 Reactive sulphur species (RSS) detection in the nucleus

Organisms' excessive generation of formaldehyde (FA) and sulfur dioxide (SO_2_) is directly linked to several ailments, such as genotoxicity, respiratory disease, and neurological abnormalities. However, the protective barrier of the cell nucleus membrane makes it challenging for fluorescent probes to investigate the correlation of FA and SO_2_ in the nucleolus regions. Ma *et al.* took this challenge and constructed probe 45 based on the benzopyryllium-dansyl FRET platform (Fig. 23, Table 1).^52^ The probe 45 was used for SO_2_ reversible sensing and recovered by FA. The sensing mechanism of SO_2_ was the interruption of the FRET process between dansyl (donor) and benzopyryllium (acceptor). The FA addition restored the FRET process because of the reversible Michael addition reaction. Probe 45 found application for quantitatively monitoring endogenous SO_2_/FA in the nucleus region of live cells and living animals.

### 8.2 Reactive nitrogen species (RNS) detection in the nucleus

**Fig. 22:**
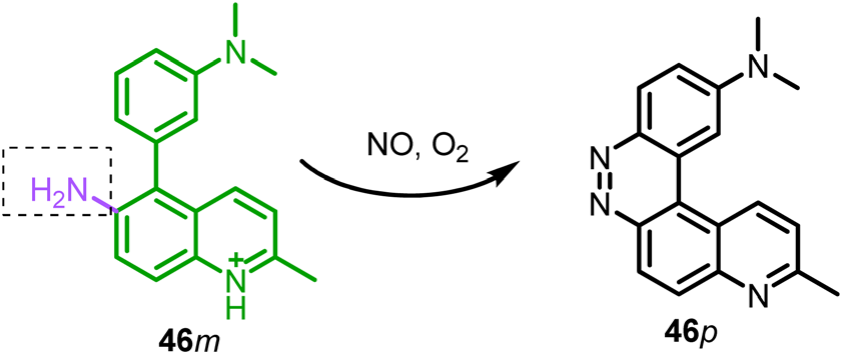
Reaction-based sensing mechanism of probe 46 for NO detection.

Nitric oxide (NO) is a significant signal molecule involved in a variety of physiological and pathological processes. Thus, to understand these processes, real-time detection of short-living NO in the biological medium is crucial. Based on their previous reports on NO detection,^167^ Li *et al.* developed probe 46 for NO detection in living cells.^168^ The fluorescence spectrum of probe 46 showed a ratiometric change in fluorescence with NO, and the plot of intensity ratio I_530_/I_424_*vs.* NO concentration was linear between 0 and 40 μM. The sensing mechanism was a reaction-based process in which 46*m* reacted with NO to generate product 46*p* (Fig. 22, Table 1). In living RAW 264.7 cells, 46*m* can detect both exogenous and endogenous NO. It's interesting to note that 46*m* and its sensing product 46*p* both show localization to the nucleus and the mitochondria, respectively. In the presence of ctDNA, 46*m* showed high sensitivity to NO (LOD = 2.8 nM). However, due to nucleus localization, 46*p* could be an excellent green-fluorescent probe for the nucleus.

### 8.3 Nucleus DNA detection

**Fig. 23:**
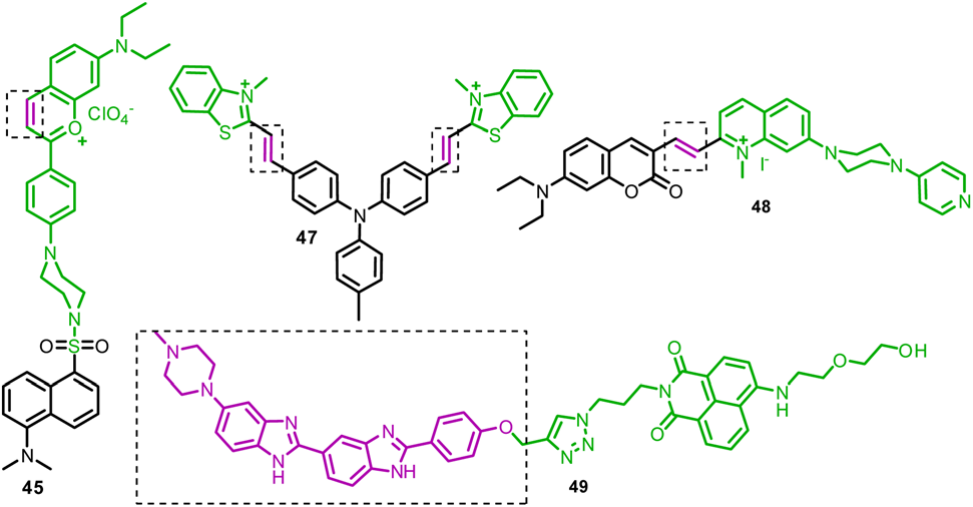
Chemical structure of nucleus-targeting ratiometric fluorescent probes 45–49.

The nucleus viscosity and G-quadruplex have several critical roles in biological processes, such as controlling gene expression, preventing tumorigenesis, *etc.* Thus, to better understand their molecular process and function, an efficient tool that can target the nucleus is in demand. Sun *et al.* developed D–π–A type probe 47 based on triphenylamine (donor) and *N*-methyl benzothiazole (acceptor and nucleus-targeting group) (Fig. 23, Table 1).^169^ With a considerable ratiometric increase in fluorescence, probe 47 revealed strong selectivity to G-quadruplex DNA and viscosity inside the nucleus. However, the notable limitation of probe 47 is that it cannot distinguish between G-quadruplex DNA and viscosity change.

Ferroptosis controls cell death by accumulating lipid peroxide-associated reactive oxygen species, which is predicted to change the shape and polarity of lipid droplets (LDs). However, there needs to be concrete proof of this. Wang *et al.* reported dual-organelle targeting (LD and nucleus) fluorescent probe 48 for monitoring cellular microenvironment polarity change (Fig. 23, Table 1).^170^ The fluorescence titration of probe 48 with ds26DNA revealed ∼11-fold increase in emission intensity at 670 nm, while emission maxima at 470 nm remained constant. From molecular modeling calculations, the selectivity of probe 48 to DNA could be due to its binding to the minor grooves of DNA through hydrogen bonding and electrostatic interactions.

Yang *et al.* developed a ratiometric fluorescent probe 49 based on a naphthalimide dye and a Hoechst (nucleus-targeting unit) (Fig. 23, Table 1).^171^ Upon gradually increasing the concentration of ctDNA, the emission maxima of probe 49 at 450 nm and 505 nm increased significantly, ascribed to the FRET process from the Hoescht to the naphthalimide unit. Probe 49's remarkable benefit is its ability to provide precise and wash-free nuclear DNA staining in living cells, including normal cells COS-7 and L02, as well as cancer cells MCF-7, SMMC-7721, and HeLa. Furthermore, probe 49 found a potential application for monitoring nucleus DNA damage induced by the anticancer drug etoposide and hydroxyl radicals.

### 8.4 Challenges

For chemical biology purposes, nucleus-targeting systems have been thoroughly investigated. However, commercially available nucleus-targeting fluorescent probes are limited to staining DNAs.^172^ Nowadays, newly developed nucleus-targeting probes are more specific to a particular analyte but are still rare. Furthermore, each developed method needs a clear explanation of organelle-specific/targeting mechanism, cellular toxicity, targeting ability in the altered environment, probe stability, response time, *etc.*

## 9 Membrane-targeting ratiometric fluorescent probes

Cytomembrane is an essential target for the study of dynamics and morphology due to the recent discovery of membrane microdomains (rafts) in cancer^173^ and viral infection.^174,175^ The cytomembrane also contributes to amyloid formation in neurodegenerative diseases.^176,177^

### 9.1 Reactive sulphur species (RSS) detection in the membrane

**Fig. 24:**
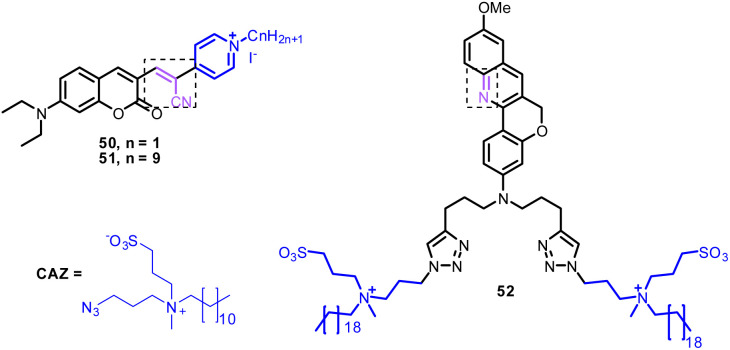
Chemical structure of membrane-targeting ratiometric fluorescent probes 50–52.

**Fig. 25:**
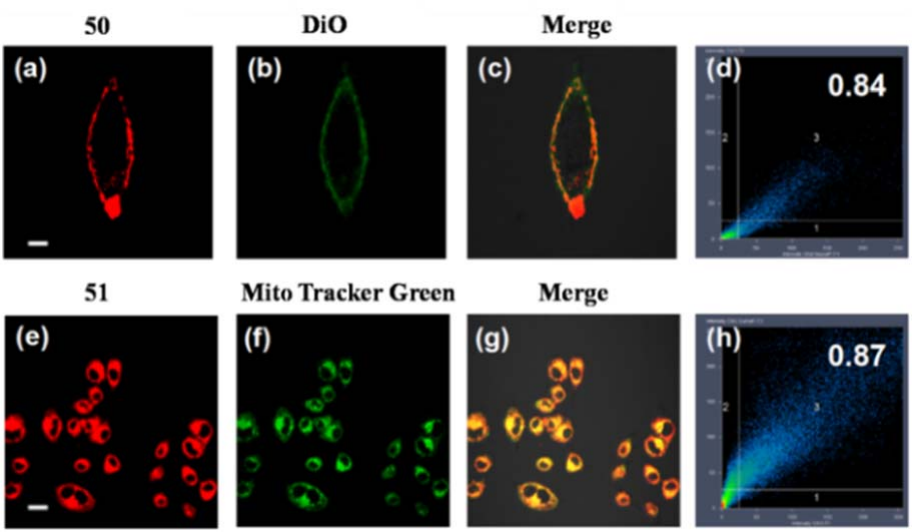
Co-localization imaging of 50 and 51. Fluorescence images of (a, e) 50 and 51 in the red channel; (b, f) MitoTracker Green in the green channel; (c, g) overlay image; (d, h) 50 and 51 intensity correlation plot. Reprinted from ref. 178, copyright 2021 Elsevier.

Zhang *et al.* developed probes 50 and 51 based on coumarin derivatives (Fig. 24, Table 1).^178^ Notably, the fluorescence spectrum of probe 50 and 51 exhibited ratiometric response with SO_2_ and visible color change of the solution from dark purple to colorless. The nucleophilic addition reaction was the sensing mechanism of probes 50 and 51 for SO_2_. From cell imaging experiments, probes 50 and 51 exhibited good cytomembrane-targeting and mitochondrion-targeting ability and can detect SO_2_ in mice. Due to the negatively charged cell inner membrane, the cationic property of probe 50 allowed it to target the cell membrane through electrostatic interactions. However, the probe 51 can selectively accumulate in mitochondria because of its positively charged nature, long alkyl chain, and appropriate hydrophobic characteristic (Fig. 25).

### 9.2 Monitoring pH changes in the membrane

The cell’s various biomolecules, pathogens, and fluids are regulated by intracellular vesicles that the plasma membrane produces.^179^ Intravesicular pH changes during trafficking depend on endosomal maturation and signaling.^180^ The cell’s various biomolecules, pathogens, and fluids are regulated by intracellular vesicles that the plasma membrane produces. Inspired by the work of Liu *et al.* on red-shifted chromenoquinoline based probes,^181^ Michelis *et al.* designed probe 52 based on chromenoquinoline for imaging the distribution and acidification of intracellular vesicles and to measure the pH of individual vesicles (Fig. 24, Table 1).^182^ From the fluorescence experiment, probe 52 underwent an increase in emission intensity upon binding with the plasma membrane (10-fold). Furthermore, probe 52 monitored the acidification of the vesicles throughout the endocytic pathway. The main limitation associated with probe 52 is that we cannot use it for long-term tracking due to the instability of the basic form.

### 9.3 Challenges

Despite several efforts in designing membrane-targeting ratiometric fluorescent probes, literature reports are rare. To date, no commercially available membrane-targeting fluorescent probes are available. In the future, researchers must consider several parameters for designing efficient membrane-targeting fluorescent probes, such as probe stability, selectivity, sensitivity, response time, orientation/location in the membrane, and detailed mechanism of interaction with the membrane.

## 10 Multi organelle-targeting ratiometric fluorescent probes

Developing a powerful molecular tool that can target other organelles simultaneously is necessary to study the relationship between different organelles.

### 10.1 Reactive sulphur species (RSS) detection in lysosome and mitochondria

**Fig. 26:**
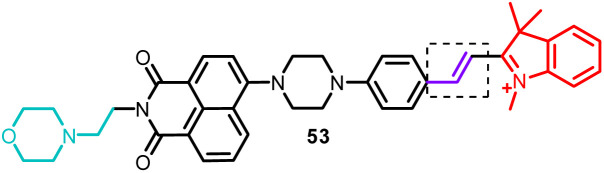
Chemical structure of dual organelle-targeting ratiometric fluorescent probe 53.

From a recent study, SO_2_ is a critical gas messenger that plays a vital role in many cellular processes, including apoptosis in lysosomes and mitochondria. Therefore, to know the relationship between lysosome and mitochondria in regulating SO_2_-related cellular activities, Kong *et al.* pioneered probe 53 (Fig. 26, Table 1).^183^ Free probe 53 showed emission at 600 nm (red fluorescence) owing to the FRET process between the naphthalimide unit and the semi-cyanine unit. Upon the addition of SO_2_, the emission intensity at 600 nm decreased. In comparison, a new emission maximum at 530 nm appeared and increased, assigned to Michael's addition reaction of SO_2_ in the semi-cyanine unit and inhibition of the FRET process. In living cells, probe 53 found an application for simultaneously detecting endogenous SO_2_ in lysosome and mitochondria by one and two-photon modes.

### 10.2 Monitoring pH fluctuation in mitochondria and lipid droplets (LDs)

**Fig. 27:**
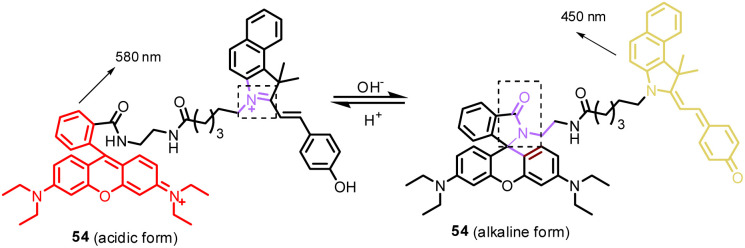
Chemical structure of probe 54 and the proposed pH sensing mechanism.

**Fig. 28:**
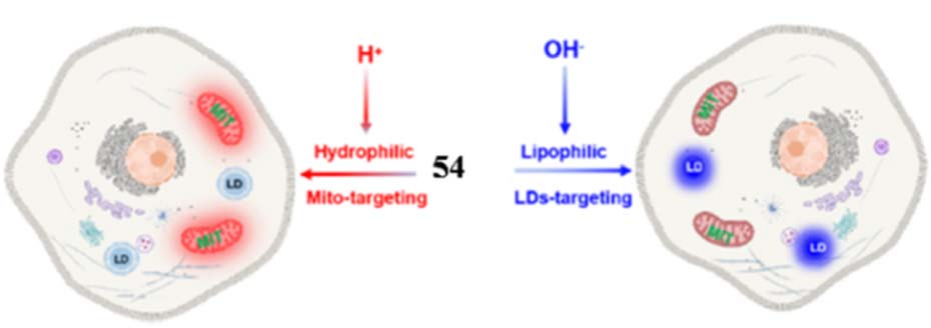
Organelle-targeting mechanism of probe 54. Reprinted from ref. 19, copyright 2022 American Chemical Society.

Understanding the fundamental connection between fluctuating mitochondrial pH and lipid droplet (LD) generation is crucial for understanding cell physiology. Bai *et al.* developed probe 54 based on hemicyanine and rhodamine dyes for selectively monitoring mitochondria and lipid droplets under different pH values through a dual-emission channel (Fig. 27 and 28, Table 1).^19^ The pH (from 2.52 to 10.50) sensing behavior of probe 54 revealed a notable decrease in the emission band at 580 nm, accompanied by the increase in a new emission peak at 450 nm, attributed to two different structural forms under acidic and basic medium. Under acidic conditions, the ring-open form of probe 54 targeted mitochondria and displayed solid red emission. In contrast, the ring-closed form of probe 54 targeted LDs and gave blue emission. Furthermore, applied probe 54 monitored the pH fluctuation in living cells in the presence of different exotic chemicals.

### 10.3 Challenges

Dual organelle-targeting probes hold great promise for elucidating the relationship between organelles and deepening our understanding of the biological processes behind the biological species. However, building molecular probes with two or more sensitive moieties is challenging. Furthermore, dual organelle-targeting probes are rare in the literature.

## 11 Conclusion and future outlook

This review article highlighted recent advances in organelle-targeting ratiometric fluorescent probes reported since 2015. In particular, we discussed synthetic fluorescent probes with potential applications in biological systems such as biological species detection, fluorescence imaging, studying physiological and pathological processes, *etc.* Additionally, we highlighted the methods utilized to construct these probes and sensing mechanisms for their response to particular species.

Nowadays, significant research has been put into developing organelle-targeting fluorescent probes to create fluorescent tools with improved resolution and sensitivity and a better understanding of the molecular mechanisms behind various biological processes. In conclusion, high selectivity, high reactivity, fast response time, low detection limit, good solubility, and organelle-targeting ability are the multiple advantages of the fluorescent probe. Currently, ratiometric probes that meet all the above properties are rare in the literature.

Researchers will always be very interested in the advancement of ratiometric probes and their commercial applications. Despite the abundance of current outcomes, the following areas require additional work in the future. First, compared to traditional one-photon fluorescence probes, two-photon fluorescent probes provide several advantages, such as deep visualization, reduced photo-toxicity, minimal light scattering, and highly bright and contrast images.^184^ Thus, the development of ratiometric probes with two-photon properties is greatly needed. Second, for *in vivo* application, near-infra-red (NIR) probes provide several advantages, such as deep tissue penetration, reduced photon scattering, and reduced photodamage to the living organism.^185^ Thus, ratiometric probes with emissions in the NIR region are in demand. Third, Reversible probes hold great promise for revealing the dynamic states of relevant analytes in various processes.^52^ Thus, developing ratiometric probes for biological species detection based on reversible reactions is essential. Fourth, probes targeting organelles such as the nucleus, membrane, lipid droplet (LD), melanosome, and Golgi apparatus are rare in the literature (Table 1). Fifth, there are several species in biological systems. However, only a limited number of species, such as ROS, RNS, RSS, metal ions, and pH, have been the subject of interest (Table 1). Sixth, most of the discussed ratiometric probes have found applications for the bio-imaging of biological species in living cells and mice. To date, no commercially available ratiometric probes are available. By taking the factors mentioned above into account in the probe design, fluorescent probes can be utilized as significant materials in the coming days, and their commercial availability will be anticipated in the future.

## Data availability

All the data were collected freely from websites such as https://scholar.google.com/ and https://www.sci-hub.se/. Microsoft Office Word 2007 was used for writing the article, while Microsoft Office PowerPoint 2007 was used for graphical presentation. ChemBioDraw was used for chemical structure drawing.

## Author contributions

Dr Manoj Kumar Goshisht and Dr Neetu Tripathi contributed equally to writing the original draft and to reviewing and editing the manuscript. Dr Goutam Kumar Patra and Dr Manohar Chaskar contributed to reviewing and editing the manuscript.

## Conflicts of interest

The authors declare that they have no known competing financial interests or personal relationships that could have appeared to influence the work reported in this paper.

## Supplementary Material
